# Glycerophosphodiester phosphodiesterase 1 mediates G3P accumulation for Eureka lemon resistance to citrus yellow vein clearing virus

**DOI:** 10.1093/hr/uhae287

**Published:** 2024-10-11

**Authors:** Ping Liao, Xue Dan, Wen Ge, Qi Zhang, Jinfa Zhao, Changyong Zhou, Yan Zhou

**Affiliations:** Integrative Science Center of Germplasm Creation in Western China (CHONGQING) Science City, Citrus Research Institute, Southwest University, Xiema Street, Beibei District, Chongqing 400712, China; National Citrus Engineering and Technology Research Center, Citrus Research Institute, Southwest University, Xiema Street, Beibei District, Chongqing 400712, China; Integrative Science Center of Germplasm Creation in Western China (CHONGQING) Science City, Citrus Research Institute, Southwest University, Xiema Street, Beibei District, Chongqing 400712, China; National Citrus Engineering and Technology Research Center, Citrus Research Institute, Southwest University, Xiema Street, Beibei District, Chongqing 400712, China; Integrative Science Center of Germplasm Creation in Western China (CHONGQING) Science City, Citrus Research Institute, Southwest University, Xiema Street, Beibei District, Chongqing 400712, China; National Citrus Engineering and Technology Research Center, Citrus Research Institute, Southwest University, Xiema Street, Beibei District, Chongqing 400712, China; Integrative Science Center of Germplasm Creation in Western China (CHONGQING) Science City, Citrus Research Institute, Southwest University, Xiema Street, Beibei District, Chongqing 400712, China; National Citrus Engineering and Technology Research Center, Citrus Research Institute, Southwest University, Xiema Street, Beibei District, Chongqing 400712, China; Integrative Science Center of Germplasm Creation in Western China (CHONGQING) Science City, Citrus Research Institute, Southwest University, Xiema Street, Beibei District, Chongqing 400712, China; National Citrus Engineering and Technology Research Center, Citrus Research Institute, Southwest University, Xiema Street, Beibei District, Chongqing 400712, China; Integrative Science Center of Germplasm Creation in Western China (CHONGQING) Science City, Citrus Research Institute, Southwest University, Xiema Street, Beibei District, Chongqing 400712, China; National Citrus Engineering and Technology Research Center, Citrus Research Institute, Southwest University, Xiema Street, Beibei District, Chongqing 400712, China; Integrative Science Center of Germplasm Creation in Western China (CHONGQING) Science City, Citrus Research Institute, Southwest University, Xiema Street, Beibei District, Chongqing 400712, China; National Citrus Engineering and Technology Research Center, Citrus Research Institute, Southwest University, Xiema Street, Beibei District, Chongqing 400712, China

## Abstract

Glycerophosphodiester phosphodiesterase 1 (GDPD1) plays an important function in the abiotic stress responses and participates in the accumulation of sn-glycerol-3-phosphate (G3P) in plants, which is key to plant systemic acquired resistance (SAR). However, the role of GDPD1 in plant responses to biotic stress remains poorly understood. This study characterized the antivirus function of the *GDPD1* gene (designated as *ClGDPD1*) from Eureka lemon. ClGDPD1 is located in the membrane and endoplasmic reticulum, where it interacts with the citrus yellow vein clearing virus (CYVCV) coat protein (CP). Compared to individually expressed ClGDPD1 or coexpressed ClGDPD1 + CP_140-326_, transiently coexpressed ClGDPD1 + CP or ClGDPD1 + CP_1-139_ significantly upregulated the key substance contents and genes expression involved in glycerophospholipid metabolism. Over-expression of *ClGDPD1* significantly facilitated the accumulation of G3P, upregulated the expression of SAR-related genes, and increased the resistance of transgenic Eureka lemon to CYVCV infection. Furthermore, exogenous glycerol treatment and over-expression of *ClGPDH* increased the G3P content and reduced CYVCV titers in plants or hairy roots. These results indicated that the enhanced resistance of *ClGDPD1* transgenic Eureka lemon to CYVCV may be due to facilitating G3P accumulation through the interaction of ClGDPD1 with CP. Our findings provide novel insights into the role of ClGDPD1 as an important regulatory center in mediating the citrus defense response to viral infections.

## Introduction

Lemon is an important fruit in the *Citrus* genus because of its complex sour flavor and vitamin C-rich juice. Lemon was introduced into Spain and North Africa between 1000 and 1200 CE and is now distributed worldwide [1]. Citrus yellow vein clearing disease is a major disease caused by the citrus yellow vein clearing virus (CYVCV) that affects lemon production in Asia and has recently spread to California [2, 3]. CYVCV-infected Eureka lemon (*Citrus limon*) exhibit vein necrosis and leaf drops, with severe cases resulting in a more than 50% reduction in lemon production [4, 5]. CYVCV belongs to *Mandarivirus* and contains six open reading frames (ORFs) [6]. To date, few studies have examined the functions of the proteins encoded by CYVCV and their interactions with host plants. A previous study showed that the coat protein (CP) encoded by ORF5 is closely associated with virus pathogenicity and symptom intensity in CYVCV-infected citrus plants [7]. Furthermore, host factors such as lemon 40S ribosomal protein S9-2 (ClRPS9-2), ascorbate peroxidase 1 (ClAPX1), and zinc finger protein DOF3.4 (ClDOF3.4) mediate host defense responses to CYVCV through interactions with CP [8–10].

Glycerophosphodiester phosphodiesterase (GDPD) is widely distributed among organisms and plays crucial roles in growth, development, and survival through lipid remodeling [11–13]. In addition, GDPD is a key enzyme in glycerophospholipid metabolism that hydrolyzes glycerophosphodiester into sn-glycerol-3-phosphate (G3P) and the corresponding alcohol [14, 15]. Previous studies have shown that G3P is a potential inducer of systemic-acquired resistance (SAR) in plants and has the ability to protect plants against bacteria and fungi [16–18]. At present, studies on how GDPD affects G3P accumulation, thereby mediating plant resistance to viruses, are missing.

We previously conducted an in-depth analysis of transcriptome raw data from Wang *et al*. [19] and found that dozens of glycerophospholipid metabolism genes, especially *ClGDPD1*, the homologous gene of *GDPD1* in Eureka lemon, were differentially expressed after Eureka lemon was infected with CYVCV. However, the role of ClGDPD1 in Eureka lemon remains unclear. Therefore, this study aims to determine the interaction between ClGDPD1 and CYVCV-CP and the function of ClGDPD1 on Eureka lemon resistance to CYVCV infection.

## Results

### Expression of *GDPD1* is highly induced by CYVCV infection

Glycerophospholipid metabolism is involved in the interactions between plants and pathogens [20, 21]. This study used real-time quantitative PCR (RT-qPCR) to test the effects of CYVCV infection on the expression of genes related to glycerophospholipid metabolism in citrus. Thirty days after CYVCV infection, the relative expression levels of glycerophospholipid metabolism-related genes (*GDPD1*, *GDPH*, *PLC2*, *PLC4*, *PLC6*, *PLD*, *PLA*, *PC*, *ACC1*, *GPAT6*, and *GPAT1*) were upregulated 1.49- to 5.87-fold in virus-free plants. Among these, the upregulation expression of *GDPD1* was the most significant and was thus selected for further study (Fig. S1a). Furthermore, the expression of *GDPD1* in virus-free sweet orange (*C. sinensis*), Meyer lemon (*C. meyerii*), *Poncirus trifoliata*, and *C. reticulata*, which are resistant to CYVCV [5, 8, 22], was 8.15-, 28.01-, 12.69-, and 6.28-fold of virus-free Eureka lemon, which is sensitive to CYVCV (Fig. S1b), respectively. Thirty days after graft inoculation with CYVCV, the expression of *GDPD1* in CYVCV-infected sweet orange, Meyer lemon, *P. trifoliata*, and *C. reticulata* was 24.21-, 98.04-, 27.03-, and 27.79-fold of virus-free Eureka lemon, respectively (Fig. S1b).

### Characterization and subcellular localization of GDPD1

ClGDPD1 has 386 amino acids (aas), approximately 40 kDa. To study the sequence conservation of GDPD1, GDPD1 protein sequences from Rutaceae species (*C. clementina*, *C. sinensis*, *P. trifoliata*, *C. australasica*, *C. grandis*, *Cycas hongheensis*, *C. ichangensis*, *C. linwuensis*, *Chirita mangshanensis*, *C. medica*, and *C. reticulata* were downloaded from the Rutaceae Pan-genome to Breeding Database http://citrus.hzau.edu.cn/; *C. limon* and *C. meyerii*) and non-Rutaceae genera (*Pistacia vera*, *Mangifera indica*, *Herrania umbratica*, *Melia azedarach*, *Theobroma cacao*, and *Malus domestica* were downloaded from the National Center for Biotechnology Information) were analyzed. The results showed that all GDPD1 sequences from *Citrus* contained a PI-PLCc_GDPD_SF superfamily domain at 48–328 aas (Fig. S2a), which hydrolyzes membrane lipid phosphatidylinositol [23]. GDPD1 sequences from Rutaceae shared high identity (>98.19%, Table S1). Compared with lemon, two mutations (Gly217 to Arg217 and Gly288 to Arg284) were found in *C. grandis*, *C. reticulate*, *C. medica*, *C. meyerii*, *C. sinensis*, and *P. trifoliata* which are resistant to CYVCV (Fig. S2a). The similarity between ClGDPD1 and non-Rutaceae GDPD1 sequences was 73.39–80.62% (Table S1). Furthermore, ClGDPD1, CmGDPD1 (from *C. meyerii*), CcGDPD1 (from *C. clementina*), and CrGDPD1 (from *C. reticulata*) were grouped into a phylogenetic tree (Fig. S2b).

**Fig. 1:**
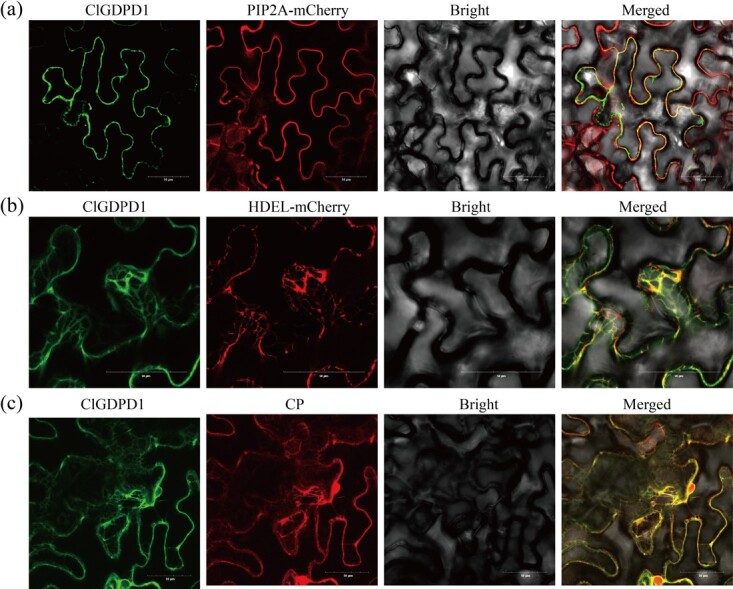
Subcellular localization of ClGDPD1. (a, b) ClGDPD1 localized in both the membrane and endoplasmic reticulum (ER) at 2 days postinfiltration (dpi). PIP2A-mCherry and HDEL-mCherry were used as the membrane and ER markers, respectively. (c) ClGDPD1 and CP colocalized in the membrane and ER at 2 dpi. Scale bars = 50 μm.

To confirm the subcellular distribution of ClGDPD1, pCHF3:ClGDPD1-GFP was constructed and coinfiltrated into *Nicotiana benthamiana* leaves with plant subcellular localization marker via agro-infiltration. Confocal microscopy image assay results showed that ClGDPD1 was present in both the membrane and endoplasmic reticulum (ER) of *N. benthamiana* cells at 2 days post-inoculation (dpi) (Fig. 1a, b). To elucidate the colocalization of ClGDPD1 and CP, pCHF3:CP-mCherry was constructed, and EHA105 culture containing pCHF3:CP-mCherry and pCHF3:ClGDPD1-GFP was infiltrated into *N. benthamiana* leaves. At 2 dpi, the green fluorescence produced by the ClGDPD1-GFP protein and the red fluorescence produced by the CP-mCherry protein overlapped at the cell membrane and ER (Fig. 1c), demonstrating that CYVCV-CP colocalized with ClGDPD1 in the membrane and ER.

### ClGDPD1 directly interacts with CP

**Fig. 2:**
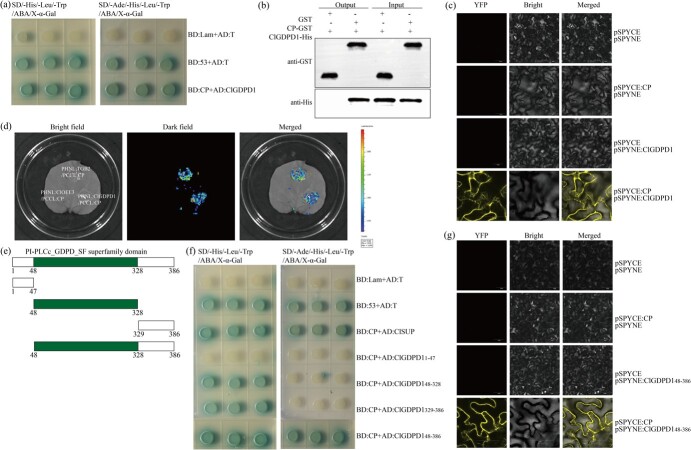
CP interacted with ClGDPD1 *in vivo* and *in vitro*. (a) The yeast two-hybrid (Y2H) assay confirmed the interaction of ClGDPD1 with CP. BD:53 + AD:T, positive control; BD:Lam + AD:T, negative control. (b) The GST pull-down assay showed ClGDPD1 interacts with CP *in vitro*. Input represents the detection of the purified protein, and output represents the protein detection after GST labeling. (c) The bimolecular fluorescence complementation (BiFC) assay shows ClGDPD1 interacts with CP. Negative controls were established using pSPYCE/pSPYNE, pSPYCE:CP/pSPYNE, and pSPYNE:ClGDPD1/pSPYCE, respectively. Scale bar = 50 μm. (d) A firefly luciferase complementation imaging (LCI) assay confirms the interaction between ClGDPD1 and CP. PHNL:TGB2/PCCL:CP, positive control; PHNL:ClOEE3/PCCL:CP, negative control. (e) Schematic representation of ClGDPD1 predicted domains and four ClGDPD1 truncated mutants. (f) The Y2H assay confirmed the interaction of truncated ClGDPD1 mutants with CP. (g) The BiFC assay confirmed that ClGDPD1_48-386_ interacted with CP. pSPYCE/pSPYNE, pSPYCE:CP/pSPYNE, and pSPYNE:ClGDPD1_48-386_/ pSPYCE were used as the negative controls. Scale bar = 50 μm.

To confirm the interaction between ClGDPD1 and CP, we performed yeast two-hybird (Y2H), pull-down, bimolecular fluorescence complementation (BiFC), and firefly luciferase complementation imaging (LCI). Firstly, AD:ClGDPD1 and BD:CP were constructed and used for the Y2H assay. The result showed that the yeasts harboring AD:ClGDPD1 and BD:CP produced blue colonies in SD/−Ade/-His/−Leu/−Trp/ABA/X-α-gal (QDO/X/A) medium, suggesting that ClGDPD1 interacted with CP (Fig. 2a). Subsequently, the C-termini of ClGDPD1 and CP were fused with His and GST tags and cloned into the BD vector to obtain BD:ClGDPD1-His and BD:CP-GST and used for the pull-down assay. The results showed that recombinant CP-GST could bind to ClGDPD1-His but GST could not bind to ClGDPD1-His (Fig. 2b). Then, BiFC revealed that CP and ClGDPD1 produced yellow fluorescent signals in the cell membrane and ER (Fig. 2c). Finally, the LCI assay was performed to further clarify this interaction in *vivo.* PHNL:ClGDPD1 and PCCL:CP were constructed and agroinfiltrated into *N. benthamiana* leaves. As shown in Fig. 2d, the PHNL:ClGDPD1/PCCL:CP injection site coinfiltrated exhibited strong luminescence, similar to that of the positive control (PHNL:TGB2/PCCL:CP injection site) at 2 dpi. These results indicate that ClGDPD1 directly interacts with CP both *in vivo* and *in vitro*, and that this interaction occurs in the membrane and ER.

To clarify the key segments of the ClGDPD1 interaction with CP, AD:ClGDPD1_1-47_ (aa residues 1–47), AD:ClGDPD1_48-328_ (aa residues 48-328), AD:ClGDPD1_48-386_ (aa residues 48-386), and AD:ClGDPD1_329-386_ (aa residues 329-386) were constructed and used for the Y2H assay. The results showed that the yeast cells cotransformed AD:ClGDPD1_48-328_/BD:CP, AD:ClGDPD1_329-386_ /BD:CP, and AD:ClGDPD1_48-386_/BD:CP produced blue colonies on SD/-His/−Leu/−Trp/ABA/X-α-gal medium, but only AD:ClGDPD1_48-386_ and BD:CP cotransformed yeast cells produced blue colonies in QDO/X/A medium (Fig. 2e, f). Furthermore, the interaction between ClGDPD1_48-386_ and CP was demonstrated via BiFC and LCI assays (Fig. 2g), and their interaction positions were the membrane and ER of *N. benthamiana*. In addition, we also investigated the key segments of the CP interaction with ClGDPD1. The Y2H and LCI assays results showed that CP_1-139_ is the crucial segment for the interaction with ClGDPD1 (Fig. S3).

### ClGDPD1 largely reduces CP expression

**Fig. 3:**
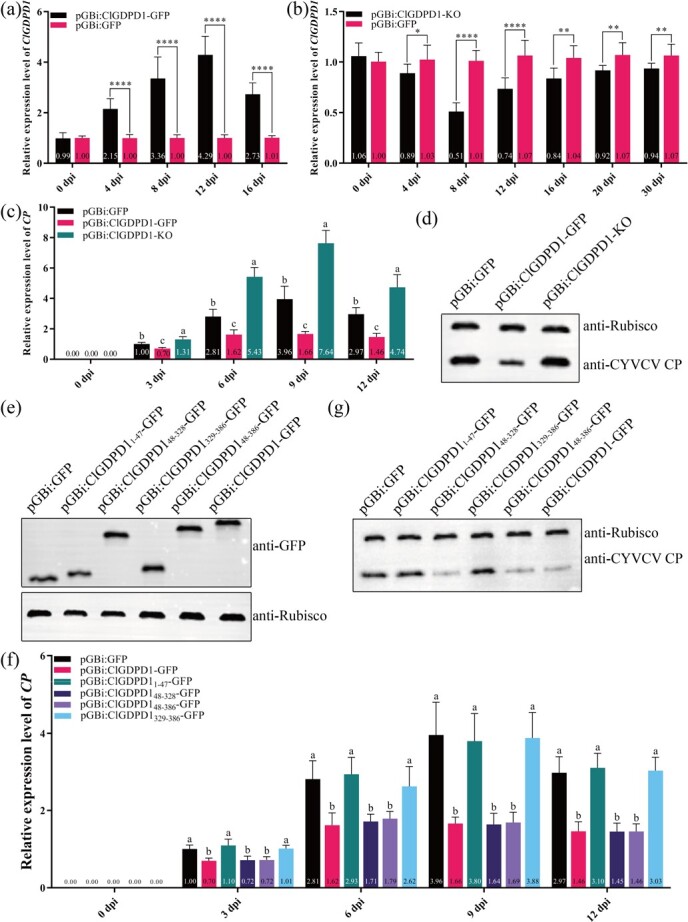
ClGDPD1 reduces CP expression. (a) The relative expression level of *ClGDPD1* in *ClGDPD1* transient over-expression Eureka lemon leaves using real-time quantitative PCR (RT-qPCR) assay. *Actin* was used as an internal reference gene, *t*-test, *n* = 9, ****p < 0.0001. (b) The relative expression level of *ClGDPD1* in *ClGDPD1*-silenced Eureka lemon leaves. *Actin* was used as an internal reference gene, *t*-test, *n* = 9, *p < 0.05, **p < 0.01, ****p < 0.0001. (c) The relative expression level of *CP* from 0 to 12 days post-infiltration (dpi) of pGBi:CP. *Actin* was used as an internal reference gene, one-way ANOVA test, *p* < 0.05 indicates significance, *n* = 9. (d) The CP content at 6 dpi was determined using western blotting (WB). (e) The ClGDPD1 truncated mutant expression was detected using WB assay at 4 dpi. (f–g) The expression of CP was assayed using RT-qPCR and WB, respectively. *Actin* was used as an internal reference gene, one-way ANOVA test, *p* < 0.05 indicates significance, *n* = 9.

To verify the function of ClGDPD1, *ClGDPD1* fused with GFP was cloned into a pGBi vector to construct pGBi:ClGDPD1-GFP, which was used to transiently express *ClGDPD1* in young Eureka lemon leaves. In addition, a 270-bp fragment of *ClGDPD1* was reverse-complementarily cloned into pGBi to generate pGBi:ClGDPD1-KO and used to silence *ClGDPD1*. Compared to the control infiltrated with pGBi:GFP, the expression of *ClGDPD1* was increased by the transient over-expression of *ClGDPD1*, with the highest expression at 12 dpi (4.29-fold) (Fig. 3a). Transient silencing of *ClGDPD1* resulted in the downregulation of *ClGDPD1*, with an expression only 50.50% that of the control at 8 dpi, and 87.85% that of the control at 30 dpi, respectively (Fig. 3b). Four days later, pGBi:CP was reinfiltrated in the Eureka lemon young leaves, which transiently expressed or silenced *ClGDPD1.* From 3 to 12 dpi, the *CP* expression in leaves transiently expressing *ClGDPD1* was significantly lower than that in control and upregulated in *ClGDPD1*-silenced leaves (Fig. 3c). The western blotting (WB) results also showed that CP content was reduced by transiently expressing *ClGDPD1* and enhanced by silencing *ClGDPD1* at 6 dpi (Fig. 3d). To exclude the effect of other *GDPDs*, the expression levels of other *GDPDs* (*GDPD3*, *GDPD4*, *GDPD6*, *GDPDL3*, and *GDPDL7*) were detected at 8 dpi. There was no significant difference in the expression levels of these *GDPDs* in *ClGDPD1*-silenced leaves compared to the controls infiltrated with pGBi:GFP (Fig. S4). These results suggested that *ClGDPD1* negatively regulates CP expression.

To identify the key areas of ClGDPD1 that perform its function to suppress CP, ClGDPD1_1-47_, ClGDPD1_48-328_, ClGDPD1_48-386_, and ClGDPD1_329-386_ were transiently expressed in Eureka lemon leaves. As shown in Fig. 3e, the four ClGDPD1 residual fragments were successfully expressed at 4 dpi. The RT-qPCR assay showed that ClGDPD1_48-328_ and ClGDPD1_48-386_ inhibited *CP* expression from 3 to 12 days after the agroinfiltration of pGBi:CP, and no significant difference was found in *CP* expression of pGBi:ClGDPD1, pGBi:ClGDPD1_48-328_, and pGBi:ClGDPD1_48-386_ agroinfiltration leaves (Fig. 3f). WB revealed that ClGDPD1_48-328_ and ClGDPD1_48-386_ inhibited the accumulation of CP (Fig. 3g). Furthermore, there was no significant difference in the transcript levels of *CP* between transiently expressed ClGDPD1_329-386_ and the controls. The results proved that ClGDPD1_48-328_ is the key segment through which ClGDPD1 inhibits CP.

### ClGDPD1 regulates the glycerophospholipid metabolism pathway

**Fig. 4:**
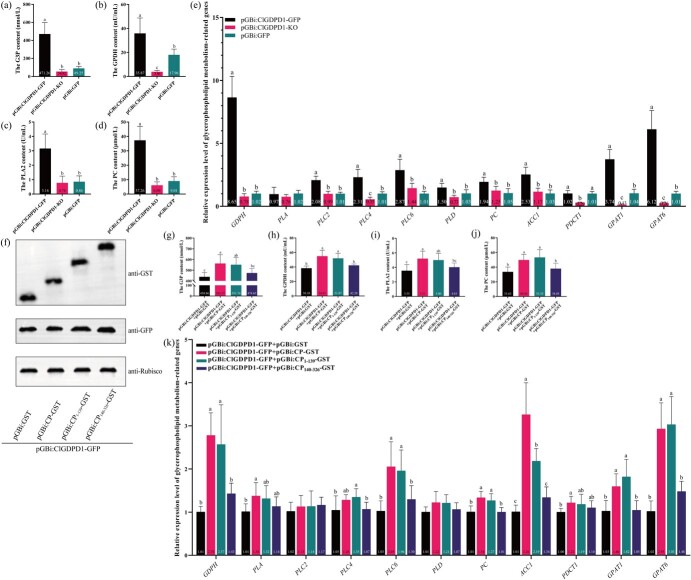
ClGDPD1 regulates the glycerophospholipid metabolism pathway. (a–d) The content of glycerol-3-phosphate (G3P), glycerol-3-phosphate dehydrogenase (GPDH), phospholipase A2 (PLA2), and phosphatidylcholine (PC) in *ClGDPD1* transient over-expression and silenced Eureka lemon leaves at 8 days past-infiltration (dpi). One-way ANOVA test, *p* < 0.05 indicates significance, *n* = 8. (e) The relative expression level of genes related to glycerophospholipid metabolism genes in *ClGDPD1* transient over-expression and silenced Eureka lemon leaves at 8 dpi. *Actin* was used as an internal reference gene, one-way ANOVA test, *p* < 0.05 indicates significance, *n* = 9. (f) ClGDPD1, ClGDPD1 + CP, ClGDPD1 + CP_1-139_ and ClGDPD1 + CP_140-326_ were transiently expressed in Eureka lemon young leaves. (g–j) The content of G3P, GPDH, PLA2, and PC in ClGDPD1, ClGDPD1 + CP, ClGDPD1 + CP_1-139_, and ClGDPD1 + CP_140-326_ transiently expressed Eureka lemon young leaves at 8 dpi. One-way ANOVA test, *p* < 0.05 indicates significance, *n* = 8. (k) The relative expression level of genes related to glycerophospholipid metabolism genes in ClGDPD1, ClGDPD1 + CP, ClGDPD1 + CP_1-139_, and ClGDPD1 + CP_140-326_ transiently expressed Eureka lemon young leaves at 8 dpi. *Actin* was used as an internal reference gene, one-way ANOVA test, *p* < 0.05 indicates significance, *n* = 9.

To further investigate the effect of ClGDPD1 on glycerophospholipid metabolism, the levels of key substances involved in glycerophospholipid metabolism, including G3P, glycerol-3-phosphate dehydrogenase (GPDH), phospholipase A2 (PLA2), and phosphatidylcholine (PC), were determined. The results showed that at 8 dpi, the contents of G3P, GPDH, PLA2, and PC in the young leaves of Eureka lemon transient over-expression of *ClGDPD1* were 5.28-, 2.00-, 3.67-, and 4.14-fold of those in the pGBi:GFP control, respectively (Fig. 4a–d). Furthermore, compared with the pGBi:GFP control, the contents of G3P, GPDH, PLA2, and PC in *ClGDPD1*-silenced Eureka lemon young leaves were decreased by 38.58%, 78.51%, 9.30%, and 32.41%, respectively (Fig. 4a–d). In addition, RT-qPCR analysis showed that most genes involved in glycerophospholipid metabolism, such as *GDPH*, *PLC4*, *GPAT6*, and *GPAT1*, were significantly upregulated by the transient over-expression of *ClGDPD1*, which was significantly downregulated in *ClGDPD1*-silenced leaves at 8 dpi (Fig. 4e).

To confirm the effect of ClGDPD1 and CP interaction on glycerophospholipid metabolism, ClGDPD1, ClGDPD1 + CP, ClGDPD1 + CP_1-139_, and ClGDPD1 + CP_140-326_ were transiently expressed in Eureka lemon young leaves using pGBi vector (Fig. 4f), respectively. At 8 dpi, the contents of G3P, GPDH, PLA2, and PC and expression levels of genes involved in glycerophospholipid metabolism (*GDPH*, *PLC6*, *PC*, *ACC1*, *GPAT6*, and *GPAT1*) in co-expressed ClGDPD1 + CP or ClGDPD1 + CP_1-139_ Eureka lemon leaves were significantly higher than those in individually expressed ClGDPD1 or ClGDPD1 + CP_140-326_ Eureka lemon leaves (Fig. 4g–k). These results indicated that the interaction between ClGDPD1 and CP may mediate glycerophospholipid metabolism.

### ClGDPD1 and G3P positively regulated the plant resistance to CYVCV

To demonstrate the function of ClGDPD1 on plant resistance to CYVCV, pGBi:ClGDPD1-GFP and pGBi:ClGDPD1-KO were used to transiently express and silence *ClGDPD1* in CYVCV-infected young Eureka lemon leaves, respectively. At 8 dpi, RT-qPCR assays showed that the CYVCV titer was reduced by 52.94% by transiently expressed *ClGDPD1* and was upregulated 3.14-fold by silencing *ClGDPD1* compared to the pGBi:GFP control (Fig. S5a). WB analysis confirmed that ClGDPD1 negatively regulated CYVCV accumulation in plants (Fig. S5b). Furthermore, G3P content in CYVCV-infected young Eureka lemon leaves transiently expressing *ClGDPD1* was 2.56-fold of the control, whereas silencing *ClGDPD1* reduced the content by 21.69% (Fig. S5c). In addition, at 30 dpi, compared with pGBi:GFP control, the CYVCV titer was upregulated 1.68-fold and G3P content was reduced 16.73% by silencing *ClGDPD1*, the CYVCV titer was reduced 22.77% and G3P content was upregulated 1.51-fold by transiently expressed *ClGDPD1* (Fig. S5d, e).

Given that G3P can be induced by the exogenous application of glycerol [24, 25], virus-free and CYVCV-infected Eureka lemon were exogenously treated with glycerol. The results showed that the G3P content in glycerol-treated virus-free Eureka lemon was 2.85-fold of the negative control (virus-free Eureka lemon sprayed with ddH_2_O), and *ClGDPD1* and SAR-related gene (*NPR1*, *PR4*, *EDS1*, *R*, *UEP1*, *HIN1*, and *PAL*) expression was significantly upregulated 20 days after glycerol treatment (Fig. S6a–c). Two months later, newly emerged leaves of glycerol-treated CYVCV-infected Eureka lemon showed quite milder symptoms than those in negative control plants sprayed with ddH_2_O (Fig. S6d). At this moment, the expression of *ClGDPD1* and G3P content after glycerol treatment was 3.25- and 3.20-fold of the negative control, respectively, and the CYVCV titer decreased by 72.28% (Fig. S6e–g). Furthermore, *ClGDPD1* was silenced in virus-free and glycerol-treated virus-free Eureka lemon young leaves, respectively, and pGBi:CP was reinfiltrated at 4 dpi. RT-qPCR results showed that the *CP* expression level in *ClGDPD1*-silenced young leaves treated with glycerol decreased by 24.51% compared to that in the *ClGDPD1*-silenced control, and SAR-related genes (*NPR1*, *PR4*, *R*, *UEP1*, *HIN1*, and *PAL*) expression were significantly upregulated (Fig. S6h–j). These results indicated that glycerol treatment can inhibit the increase in CP expression caused by silencing *ClGDPD1* by compensating for the decrease in G3P levels.

To further confirm the connection between G3P involved in host defense against CYVCV, *ClGDPD1* was silenced in CYVCV-infected Eureka lemon young leaves treated with glycerol or H_2_O, respectively. The CYVCV titer in *ClGDPD1*-silenced CYVCV-infected young leaves treated with glycerol was 39.60% and 62.38% of that in *ClGDPD1*-silenced CYVCV-infected leaves treated with H_2_O at 8 dpi and 30 dpi (Fig. S7a), respectively. The G3P content in *ClGDPD1*-silenced CYVCV-infected young leaves treated with glycerol was 2.07- and 1.67-fold of that in *ClGDPD1*-silenced CYVCV-infected leaves treated with H_2_O at 8 dpi and 30 dpi (Fig. S7b), respectively.

In addition, a previous study suggested that GPDH can mediate G3P synthesis [26]. In this study, pLGN:ClGPDH was constructed to establish the Eureka lemon *ClGPDH* hairy root transformation system, as Xiao *et al*. described previously [27]. Sixty days later, the transformation of the hairy root was characterized, and positive samples were used in subsequent experiments (Table S2). As shown in Fig. S8, the G3P content and CYVCV titer in the *ClGPDH* transgenic hairy root were 2.12-fold and 50.50% of those of the control, respectively. Collectively, the above results indicate that G3P mediates the immune response of Eureka lemon to resist CYVCV infection.

### Resistance to CYVCV in *ClGDPD1* transgenic lemon through mediating G3P accumulation

**Fig. 5:**
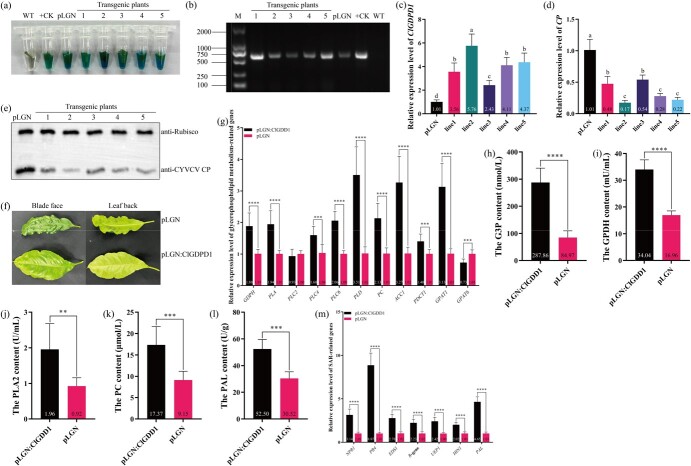
*ClGDPD1*-OE transgenic Eureka lemon resistance to CYVCV through mediating glycerol-3-phosphate (G3P) accumulation. (a) *ClGDPD1*-OE transgenic lines were validated using β-glucuronidase histochemical staining. Tube 1, negative control, tube 2, positive control; tube 3, pLGN transgenic plant; tubes 4–8, transgenic lines 1–5. (b) *ClGDPD1*-OE transgenic lemon plants were confirmed using a PCR assay. M, DL2000 marker; transgenic 1-5, *ClGDPD1* transgenic Eureka lemon lines 1–5; pLGN, pLGN transgenic Eureka lemon; +CK, positive control; WT, negative control. (c) The relative expression level of *ClGDPD1* in transgenic plants. One-way ANOVA test with *p* < 0.05, *n* = 9. (d, e) The CYVCV titer was quantified using real-time quantitative PCR (RT-qPCR) and western blotting (WB) analysis at 60 days after inoculation (dpi). One-way ANOVA test, *p* < 0.05, and *n* = 9 for RT-qPCR. Anti-CYVCV CP was used for the WB assay. (f) The symptoms induced by CYVCV in the *ClGDPD1* and pLGN transgenic Eureka lemon were photographed 1 year after inoculation. (g, m) The relative expression levels of genes related to glycerophospholipid metabolism and systemic acquired resistance (SAR). *Actin* was used as an internal reference gene, *t*-test, *n* = 9, ***p < 0.001, ****p < 0.0001 for the RT-qPCR assay. (h–k) The content of G3P, GPDH, PLA2, and PC in *ClGDPD1* transgenic Eureka lemon. *t*-test, **p < 0.01, ***p < 0.001, ****p < 0.0001, *n* = 8. (l) The content of phenylalanine ammonia lyase (PAL) in *ClGDPD1* transgenic Eureka lemon. *t*-test, ***p < 0.001, *n* = 5.

To further clarify the impact of *ClGDPD1* on Eureka lemon response to CYVCV infection, pLGN:ClGDPD1 containing a 35S promoter was constructed and used for Eureka lemon genetic transformation. Five *ClGDPD1* over-expression (*ClGDPD1*-OE) transgenic lines were obtained via GUS staining and PCR verification (Fig. 5a, b). The RT-qPCR assay showed that *ClGDPD1* expression in these transgenic lines was 3.52-, 5.70-, 2.41-, 4.07-, and 4.33-fold of the pLGN control (Fig. 5c), respectively. The transgenic lines were propagated by grafting the buds onto 2-year-old virus-free Eureka lemon seedlings. Sixty days after graft inoculation with CYVCV, transgenic lines 2 and 5 showed the lowest CYVCV titer (16.83% and 21.78%, respectively) (Fig. 5d, e). WT plants showed typical symptoms, such as vein clearing and vein necrosis, approximately 30 days after CYVCV graft inoculation and the symptoms gradually worsened. Comparatively, the *ClDGDPD1* transgenic Eureka lemon only showed mild leaf chlorotic spot symptoms within 1 year (Fig. 5f).

To further elucidate the ClGDPD1-mediated defense mechanism against CYVCV, the accumulation of glycerophospholipid metabolism-related substances and expression of related pathway genes in virus-free *ClGDPD1*-OE transgenic plants were examined. As shown in Fig. 5g, in *ClGDPD1*-OE transgenic plants, the relative expression levels of the glycerophospholipid metabolism-related genes such as *GDPH*, *PLC4*, *PLC6*, *PLD*, *PLA*, *PC*, *ACC1*, *PDCT1*, and *GPAT1* were 1.86-, 1.54-, 2.06-, 3.44-, 1.92-, 2.11-, 3.21-, 1.39-, and 3.10-fold those of the pLGN control, respectively. The contents of glycerophospholipid metabolism-related substances G3P, GPDH, PLA2, and PC in *ClGDPD1*-OE transgenic plants were 3.39-, 2.01-, 2.13-, and 1.90-fold of those in the control (Fig. 5h–k). As G3P is an inducer of SAR [28], so the phenylalanine ammonia-lyase (PAL) content and the expression of SAR-related genes in the *ClGDPD1*-OE transgenic Eureka lemon were detected. The results showed that the PAL content in *ClGDPD1*-OE was 1.72-fold that of the control, and the expression of SAR-related genes (*NPR1*, *PR4*, *EDS1*, *R*, *UEP1*, *HIN1,* and *PAL*) was significantly upregulated (Fig. 5l–m). As shown in Fig. S9, compared with virus-free *ClGDPD1*-OE transgenic plants, the contents of G3P, GPDH, PLA2, and PC and expression levels of genes involved in glycerophospholipid metabolism (*GDPH*, *PLA*, *PLC4*, *PLD*, *ACC1*, *GPAT1*, and *GPAT6*) and SAR (*NPR1*, *PR4*, *EDS1*, *R*, *UEP1*, and *PAL*) in CYVCV-infected *ClGDPD1*-OE transgenic plants were significantly up-regulated. These results indicated that ClGDPD1-mediated G3P accumulation, upregulated SAR-related genes expression, and enhanced Eureka lemon resistance to CYVCV.

### Transcriptome profiling of transgenic plants further reveals the molecular mechanism of ClGDPD1 resistance to CYVCV

A transcriptomic analysis was performed to further elucidate the functional roles of ClGDPD1 in response to CYVCV infection in Eureka lemon. Young leaves from *ClGDPD1* transgenic plants and pLGN plants were collected 60 days after inoculation with CYVCV and subjected to RNA-seq analyses. The raw data were filtered and mapped to the *C. sinensis* reference genome. As shown in Table S3, in total, 38 859 534–47 360 184 total reads and 19 429 767–23 680 092 clean reads were obtained, with more than 86.47%–89.24% of reads mapped to the reference genome and more than 82.54% uniq mapped. Thus, these transcriptome data are reliable and help in exploring the mechanism of CYVCV resistance in *ClGDPD1* transgenic plants.

**Fig. 6:**
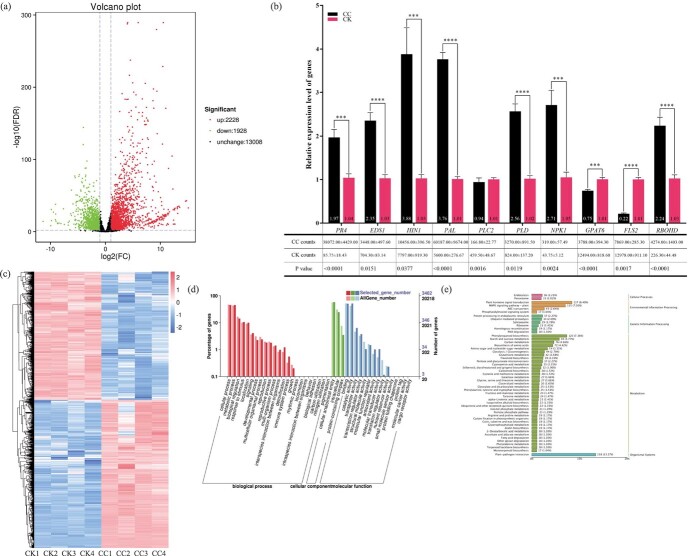
Transcriptome analysis of CYVCV-infected *ClGDPD1* transgenic Eureka lemon. (a) Volcano plot of the differentially expressed genes (DEGs) in pLGN (CK) vs. pLGN:ClGDPD1 (CC). (b) The relative expression levels of the DEGs were assayed using real-time quantitative PCR (RT-qPCR). *Actin* was used as an internal reference gene, *t*-test, *n* = 9; ***p < 0.001, ****p < 0.0001. (c) Gene ontology enrichment analysis in CK vs. CC. (d, e) Kyoto Encyclopedia of Genes and Genomes enrichment analysis between CC and WT plants.

As shown in Fig. 6a, a total of 4156 differentially expressed genes (DEGs) were identified from pLGN (CK) vs. pLGN:ClGDPD1 (CC), of which 2228 were upregulated and 1928 were downregulated. To verify the DEGs identified in the RNA-seq analysis, 10 DEGs related to glycerophospholipid metabolism, SAR, and mitogen-activated protein kinases (MAPK) were selected for expression analysis using RT-qPCR. The expression level of *PLC2* determined using RT-qPCR was downregulated compared to that determined using transcriptome analyses, but the difference was not significant. All other genes displayed similar expression patterns to the RNA-seq results (Fig. 6b).

Cluster analysis of the samples showed that the expression level of the DEGs was reproducible in all four replicates (Fig. 6c). Gene ontology (GO) enrichment analysis revealed the “metabolic process” and “cellular process” of biological process, “cellular anatomical entity” of cellular component, and “catalytic activity” and “binding” of molecular function (Fig. 6d). To further reveal differences among the metabolic pathways of DEGs and understand their functions, the Kyoto Encyclopedia of Genes and Genomes enrichment analysis was utilized. Multiple DEGs were enriched in glycerophospholipid metabolism (Fig. S10). Furthermore, we found that the top 4 pathways enriched for DEGs were “plant-pathogen interaction”, “phenylpropanoid biosynthesis”, “MAPK signaling pathway-plant”, and “plant hormone signal transduction” (Fig. 6e). The relevant DEGs were annotated as shown in Table S4. The DEGs annotated as *ERF1*, *ERF2*, *WRKY1*, *WRKY2*, *WRKY22*, *WRKY29*, and *EDS1* were upregulated and related to plant–pathogen interactions, among them, *WRKY22* and *WRKY29* were also key genes for MAPK. The up- and downregulated DEGs annotated *PR1* and *MYC2* were related to MAPK and plant hormone signal transduction. During plant hormone signal transduction, the DEGs annotated with *EBF1*, *EBF2*, *GID1*, and *BSK* were upregulated.

## Discussion

Identifying the role of plant proteins and their interaction with viruses is crucial for elucidating the virus pathogenic mechanism, controlling the development and spread of plant diseases [29, 30]. Recent studies have shown that CP interacts with ClRPS9-2, ClDOF3.4, and ClAPX1 to regulate lemon resistance to CYVCV [8–10]. In this study, the glycerophospholipid metabolism-related protein ClGDPD1 interacted with CP *in vivo* and *in vitro*. The 48–386 aas of ClGDPD1 is key segment for its interaction with CP, and the 48–328 aas of ClGDPD1 can suppress CP accumulation in plants. The results provide a reference for elucidating the regulatory network of CYVCV–host interactions, indicating that CP is a multifunctional protein that plays an important role in virus-host interactions and lemon responses to CYVCV infection.

*GDPD1* is a small gene family with a highly conserved PI-PLCc_GDPD_SF superfamily domain that plays an important role in plant responses to abiotic stress, including maintenance of cellular phosphate homeostasis, acclimation to Pi limitation, mediation of root hair development, and encoding an active GDPD enzyme [15, 31, 32]. We found that ClGDPD1 is a positive regulator of plant defense responses and can suppress the accumulation of CP and CYVCV and reduce the development of CYVCV symptoms in Eureka lemons. These results enrich the understanding of the function of GDPD1 in plant responses to biological stress and provide a promising candidate target for breeding strategies to improve lemon resistance to CYVCV.

Previous studies have shown that exogenous glycerol treatment and over-expression of homologous *GPDH* enhanced G3P content activating resistance to fungi and bacteria, such as *Phytophthora capsici*, *Colletotrichum higginsianum*, *Xanthomonas oryzae* pv*. oryzae*, and *Magnaporthe oryzae* [33–35]. However, few studies have investigated the function of G3P against plant viruses. The present study showed that exogenous glycerol treatment and over-expression of *ClGPDH* could inhibit CYVCV titer in plants or hairy roots by increasing G3P accumulation. Further study demonstrated that the ability of *ClGDPD1* to improve Eureka lemon resistance to CYVCV was also related to its positive regulation of G3P content. Therefore, we will further evaluate the role of glycerol treatment in the prevention and control of CYVCV infection and symptom development, which may provide a potential approach to reduce the loss of CYVCV and CYVCV transmission in the field in the future.

G3P is a well-known mobile regulator of SAR [18, 36] and can induce SAR and enhance the resistance of *Arabidopsis thaliana* to *C. higginsianum* [35]. The resistance of wheat to powdery mildew mediated by G3P is regulated by plant hormone cross-talk genes that are closely related to SAR [16]. Furthermore, high G3P level can produce reactive oxygen species (ROSs), induce the expression of many pathogenesis-related genes, and enhance the resistance of *T. cacao* to *P. capsici* [24]. In this study, the content of PAL, an SAR marker substance [37], was increased and SAR-related genes were upregulated by the over-expression of *ClGDPD1* and increased G3P content. Future studies should explore the role of SAR in plant responses to CYVCV infection.

RNA-seq assay of *ClGDPD1* transgenic lemon revealed that many DEGs are enriched in glycerophospholipid metabolism, but most enriched in “plant-pathogen interaction”. DEGs annotating WRKY family genes (*WRKY1*, *WRKY2*, *WRKY22*, and *WRKY29*), ethylene responsive factor (*ERF*), and enhanced disease susceptibility 1 (*EDS1*), which belong to the plant–pathogen interaction pathway and play an important role in plant responses to biological stress, were upregulated [38–41]. These DEGs were also related to plant hormone signal transduction and MAPK pathways, which were the third and fourth DEGs enrichment signaling pathways, respectively. Previous studies have shown that jasmonic acid, ethylene, and salicylic acid signal transduction in these two pathways are closely related to plants responses to pathogens [42, 43]. MAPK cascades are involved in ROS generation, defense gene activation, phytoalexin biosynthesis, biosynthesis and signaling of plant stress and defense hormones, and hypersensitive response cell death to enhance plant resistance to viruses, such as bamboo mosaic virus, barley yellow striate mosaic virus, beet black scorch virus, and other viruses [44–47]. In summary, the MAPK and plant hormone signaling pathways should be the focus of future studies on the interaction between CYVCV and host factors.

*Agrobacterium tumefaciens*-mediated citrus genetic transformation has been used to improve citrus resistance to *Candidatus* Liberibacter asiaticus, *Xanthomonas citri* pv. *citri*, *Citrus tristeza virus*, *Citrus psorosis virus*, and CYVCV [8, 48–50]. However, this traditional technology is complicated, time-consuming, and has a low genetic transformation rate. To address this shortcoming, *Agrobacterium rhizogenes*-mediated hairy root transformation system has been developed and widely used for the genetic transformation of grape, rice, cotton, lychee, and other plant species [51–54]. Xiao *et al*. transferred an *auxin* reporter gene into *P. trifoliata* via *A. rhizogenes*-mediated hairy root transformation [55], and the efficient expression of exogenous genes for up to 4 months [56, 57]. In this study, this transformation system was used to study the function of G3P in Eureka lemon resistant to CYVCV. This method provided an effective way to rapidly identify the functions of plant candidate genes. In the future, we will construct *ClGPDH* transgenic citrus to enrich a new citrus germplasm resistant to CYVCV and further explore the mechanism of citrus resistance to yellow vein disease.

In conclusion, the interaction between ClGDPD1 and CP significantly upregulated G3P content and genes expression involved in glycerophospholipid metabolism. Over-expression of *ClGDPD1* in *ClGDPD1* transgenic lemon decreases CYVCV content and facilitates the accumulation of G3P. This study provides a foundation for elucidating the mechanisms of citrus resistance in CYVCV.

## Materials and methods

### Plant materials, virus source, and primers

Virus-free *Nicotiana benthamiana*, Eureka lemon (*C. limon*), transgenic Eureka lemon, sweet orange (*Citrus sinensis*), Meyer lemon (*Citrus meyerii*), *P. trifoliata*, and *Citrus reticulata* seedlings and Eureka lemon infected with CYVCV CQ (KX156736.1) were cultivated in a greenhouse at 25 ± 3°C. All primers used in this study are shown in Table S5.

### Characterization of GDPD1

The amino acid sequences of GDPD1 (*Citrus australasica* (Egl276330.1), *Citrus clementina* (XP_006447005), *Citrus grandis* (Cg2g010320.1), *Citrus hongheensis* (Chh232450.1), *Citrus ichangensis* (Cic186990.1), *C. limon* (WKB17708.1), *Citrus linwuensis* (LW231400.1), *Citrus mangshanensis* (Cms123180.1), *Citrus medica* (Cme125720.2), *C. reticulata* (Cre2g_022810.1), *C. sinensis* (KAH9791820.1), *Citrus meyerii* (PP895201), *P. trifoliata* (Pt2g009590.1), *Herrania umbratica* (XP_021297951.1), *M. domestica* (XP_008341262.2), *M. indica* (XP_044502999.1), *M. azedarach* (KAJ4729513.1), *P. vera* (XP_031271470.1), and *T. cacao* (XP_007031952.2)) were aligned using the Jalview and DNAMAN softwares [58, 59]. The alignment obtained was used to construct a phylogenetic tree using the neighbor-joining method using the MEGA package [60].

### Subcellular localization of ClGDPD1

Procedures previously described by Zeng *et al*. were followed [8]. The coding sequence of *ClGDPD1* was fused with GFP and cloned into an *Xba*I/*Xma*I digested pCHF3 vector to obtain pCHF3:ClGDPD1-GFP. The construct was introduced into *Agrobacterium* EHA105 and coinfiltrated into *Nicotiana benthamiana* with subcellular localization markers PIP2A-mCherry (membrane) and HDEL-mCherry (ER), respectively [61, 62]. Furthermore, the pCHF3:CP-mCherry vector was constructed and coinfiltrated *Nicotiana benthamiana* with pCHF3:ClGDPD1-GFP via agroinfiltration. Fluorescent signals were then observed under an FV3000 scanning confocal microscope (FV3000, Olympus, Japan).

### Y2H assay

The Y2H system (Weidi, China) was utilized to investigate the interactions between ClGDPD1 and CP. *ClGDPD1* and *CP* were ligated into the *Bam*HI/*Eco*RI sites of the pGADT7 (AD) and pGBKT7 (BD) vectors to generate AD:ClGDPD1 and BD:CP, respectively. The Y2H assay was performed, as described previously [63].

### Pull down assay

His and GST tags were fused to the C-termini of ClGDPD1 and CP, respectively, and cloned into the pGBKT7 (BD) vector to obtain BD:ClGDPD-His and BD:CP-GST. ClGDPD-His and CP-GST fusion proteins were expressed in the TNT®T7/SP6 Coupled Wheat Germ Extract System (Promega, USA) and pull-down assays were performed, as previously described [64].

### BiFC assay

*ClGDPD1* and *CP* were amplified and cloned into *Bam*HI/*Sal*I double-digested pSPYNE and pSPYCE vectors (Puint, China), respectively, to generate pSPYNE:ClGDPD1 and pSPYCE:CP. These recombinant plasmids were agro-infiltrated *N. benthamiana* leaves [65]. Scanning confocal microscope was used to observe the fluorescence signals at 2 dpi [8].

### LCI assay

Cloning *ClGDPD1* and *CP* into the *Sal*I/*Kpn*I sites of the PHNL-P14 and PCCL-P9 vectors results in PHNL:ClGDPD1 and PCCL:CP, respectively. The *Agrobacterium* carrying these constructs was coinfiltrated into *Nicotiana benthamiana* leaves. The infiltrated plants were cultured for 48 h, after which D-Luciferin (1 mM, Macklin, China) was sprayed onto the infiltrated leaves. The leaves were imaged using a Molecular Imaging System for Living Animals and Plants (PerkinElmer, USA) [66].

### Transient over-expression and suppression of *ClGDPD1* in Eureka lemon

To transiently express and suppress *ClGDPD1* in Eureka lemon leaves, *ClGDPD1* was fused with GFP and cloned into a pGBi vector to obtain the *ClGDPD1* gene over-expression recombinant plasmid pGBi:ClGDPD1-GFP. The 270-bp fragment of *ClGDPD1* was reverse complementarily cloned into a pGBi vector to obtain the *ClGDPD1* gene silencing recombinant plasmid pGBi:ClGDPD1-KO. These plasmids were, respectively, infiltrated into the young leaves of Eureka lemon via agroinfiltration.

### Eureka lemon transformation and resistance evaluation

*Agrobacterium* strain EHA105 containing the pLGN:ClGDPD1 vector was used in the Eureka lemon transformation system. Subsequently, *Agrobacterium*-mediated transformation of the epicotyl segments of Eureka lemon was performed [8]. The transgenic *ClGDPD1* Eureka lemon was confirmed by GUS staining and RT-PCR, and the positive buds were grafted onto 2-year-old virus-free Eureka lemon seedlings. Six months later, the resistance of transgenic plants to CYVCV was evaluated [8]. Sixty days after inoculation with CYVCV, fresh leaves were collected for CYVCV quantification and sent to Biomarker Technologies Co. Ltd. (Beijing, China) for Illumina sequencing.

### Glycerol treatment

To increase the G3P level in Eureka lemon plants and explore their ability to resist CYVCV, virus-free and CYVCV-infected Eureka lemon plants were treated with a 4% glycerol solution prepared in sterile water containing 0.04% Silwett L-77 [24]. Glycerol was sprayed every 3 days for a total of eight treatments.

### Eureka lemon hairy root transformation to the expression of *ClGPDH*

We further increased the G3P content and explored its effect on CP expression. *ClGPDH* was cloned into a pLGN vector to construct pLGN:ClGPDH and used to establish an *A. rhizogenes*-mediated genetic transformation system using CYVCV-infected hairy roots [27].

### Physiological measurements

The contents of glycerophospholipid metabolism-related substances (G3P, GPDH, PLA2, and PC) and SRA-related substance PAL were determined using corresponding kits (Sinobestbio, China).

### Gene expression analysis and WB

Total RNA and RT-qPCR were performed as described by Shen *et al*. [62]. The 2^−ΔΔCt^ method was used to perform the gene expression calculation [67]. WB assay was performed as Zeng *et al*. described [8].

### Statistical analysis

GraphPad Prism 9.3.1 was used to analyze the data. *t*-test was utilized to analyze differences between two groups, and one-way ANOVA test was employed to assess differences among three or more groups [68].

## Acknowledgments

We sincerely thank Professors Xiaochun Zhao (Southwest University, Chongqing, China) and Xiuping Zou (Southwest University, Chongqing, China) providing pGBi and pLGN plasmids, respectively, and Professor Jianmin Zhou (University of Chinese Academy of Sciences, Beijing, China) for the PHNL-P14 and PCCL-P9 plasmids. This research was partially supported by China Agriculture Research System of MOF and MARA (CARS-26-05B) and Overseas Expertise Introduction Project for Discipline Innovation, Ministry of Education of the People’s Republic of China (CN) (B18044).

## Author Contributions

P. L., Y. Z., and C. Z. designed the experiments. P. L., X. D., W. G., and Q. Z. performed the experiments. P. L., X. D., and J. Z. analyzed the data. P. L. wrote the manuscript. P. L., Y. Z., and C. Z. revised and polished the manuscript. All authors contributed to the article and approved the submitted version.

## Supplementary Material

Web_Material_uhae287

## Data Availability

The authors confirm that all the experimental data may be found in the main text and the supplemental data.
