# Supplementary material for: Glycerophosphodiester phosphodiesterase 1 mediates G3P accumulation for Eureka lemon resistance to citrus yellow vein clearing virus
Source: Hortic Res. 2024 Oct 11;12(1):uhae287. doi: 10.1093/hr/uhae287 (PMC11775586; doi:10.1093/hr/uhae287)
Supplement: Web_Material_uhae287 [file web_material_uhae287.zip › Supplementary.docx]

**Supplemental figures**


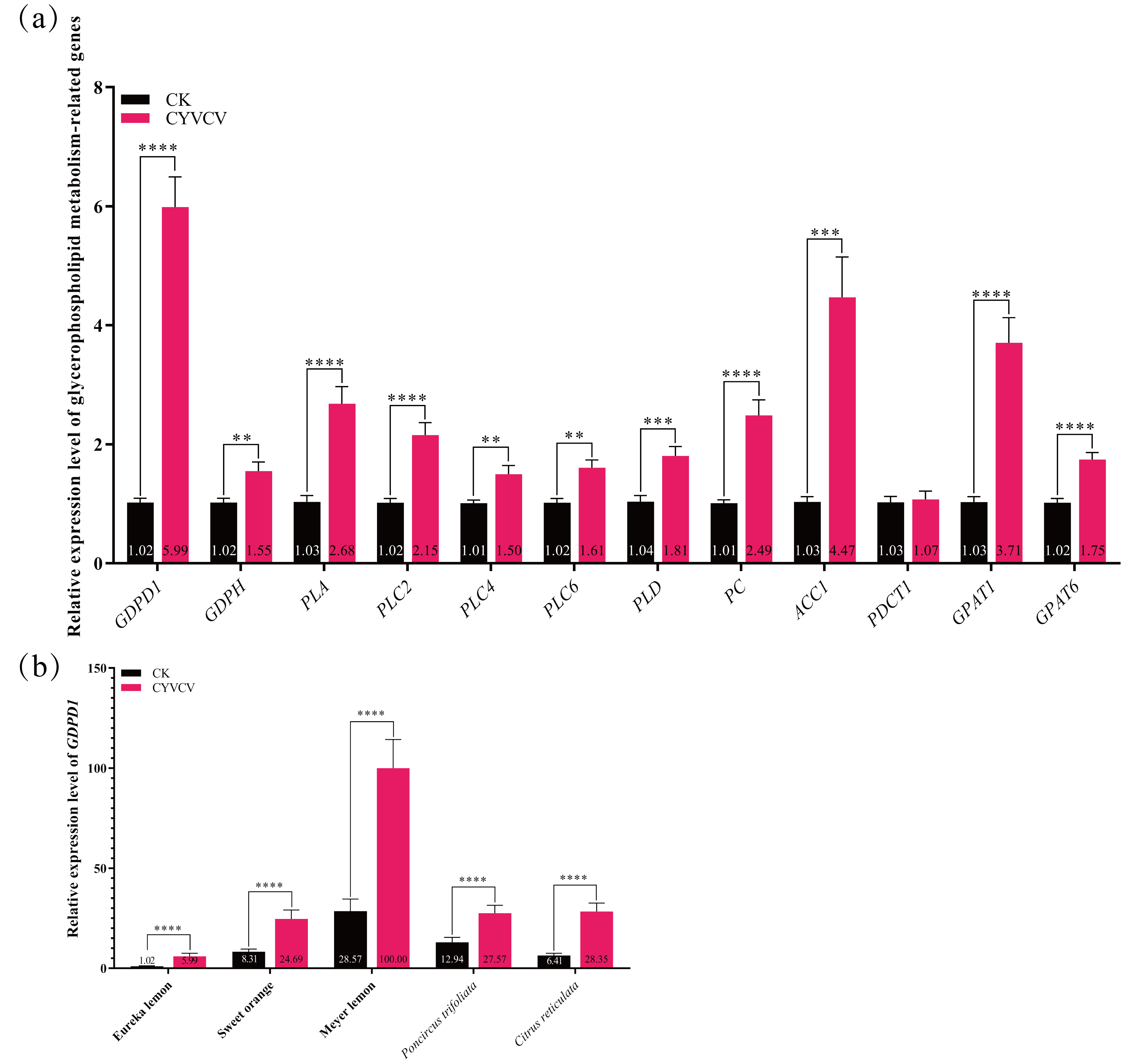


**Fig. S1 *GDPD1* is highly induced by CYVCV infection**. (a) The relative expression level of glycerophospholipid metabolism-related genes at 30 days after graft-inoculation with CYVCV. (b) The relative expression level of *GDPD1* in Eureka lemon, sweet orange, Meyer lemon, *Poncircus trifoliata*, and *Citrus reticulata* at 30 days after graft-inoculated with CYVCV. *Actin* was used as an internal reference gene, t-test, n = 9; ***p*<0.01; ****p*<0.001; *****p*<0.0001.

**
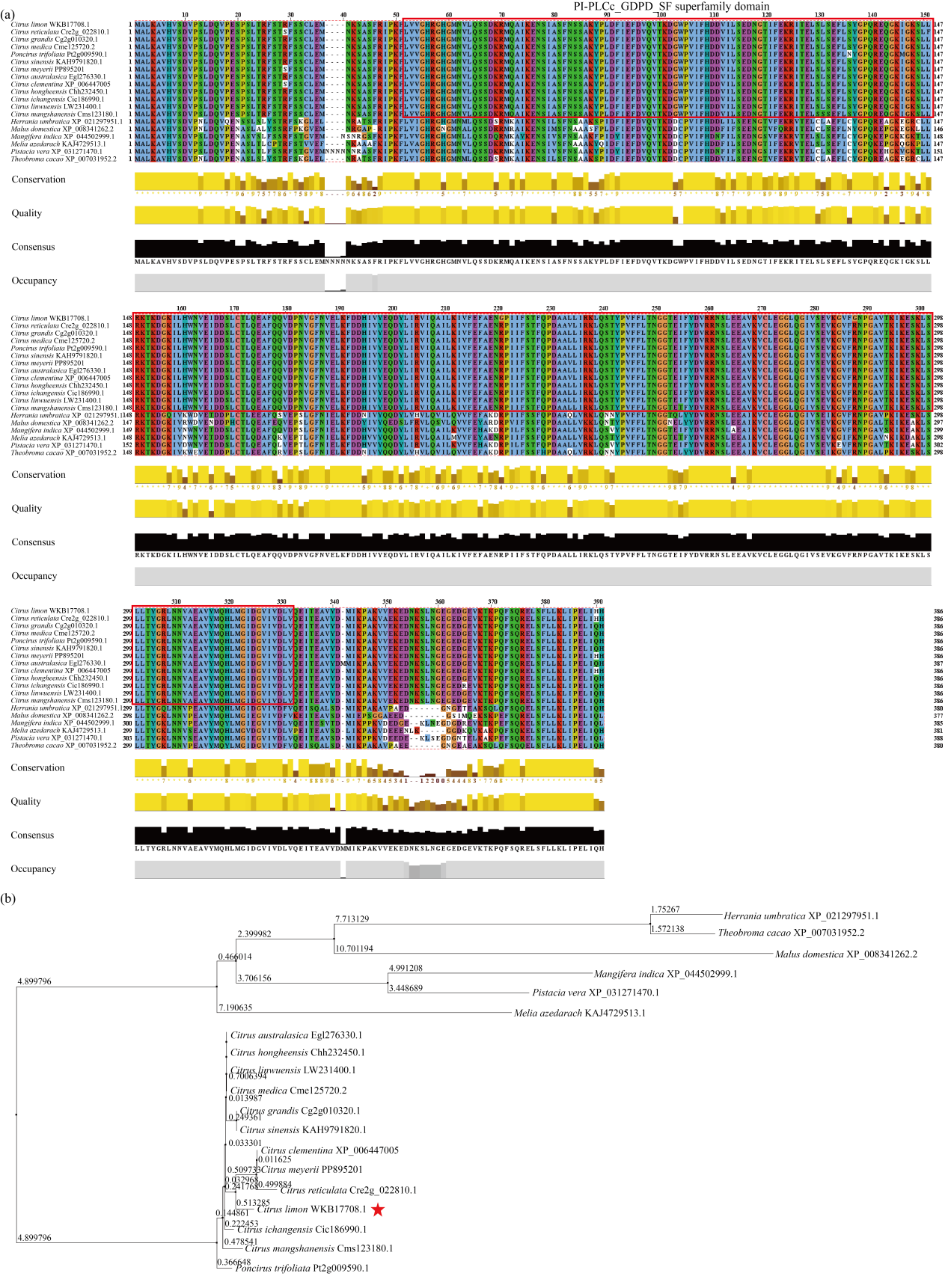
Fig. S2 Characterization and phylogenetic analyses of GDPD1.** (a) The PI-PLCc_GDPD_SF superfamily domain is highly conserved across the Rutaceae species and non-Rutaceae species. (b) Phylogenetic tree showing the full-length amino acid sequences for the GDPD1 proteins. The phylogenetic tree was constructed using the neighbour-joining method with Jalview software.


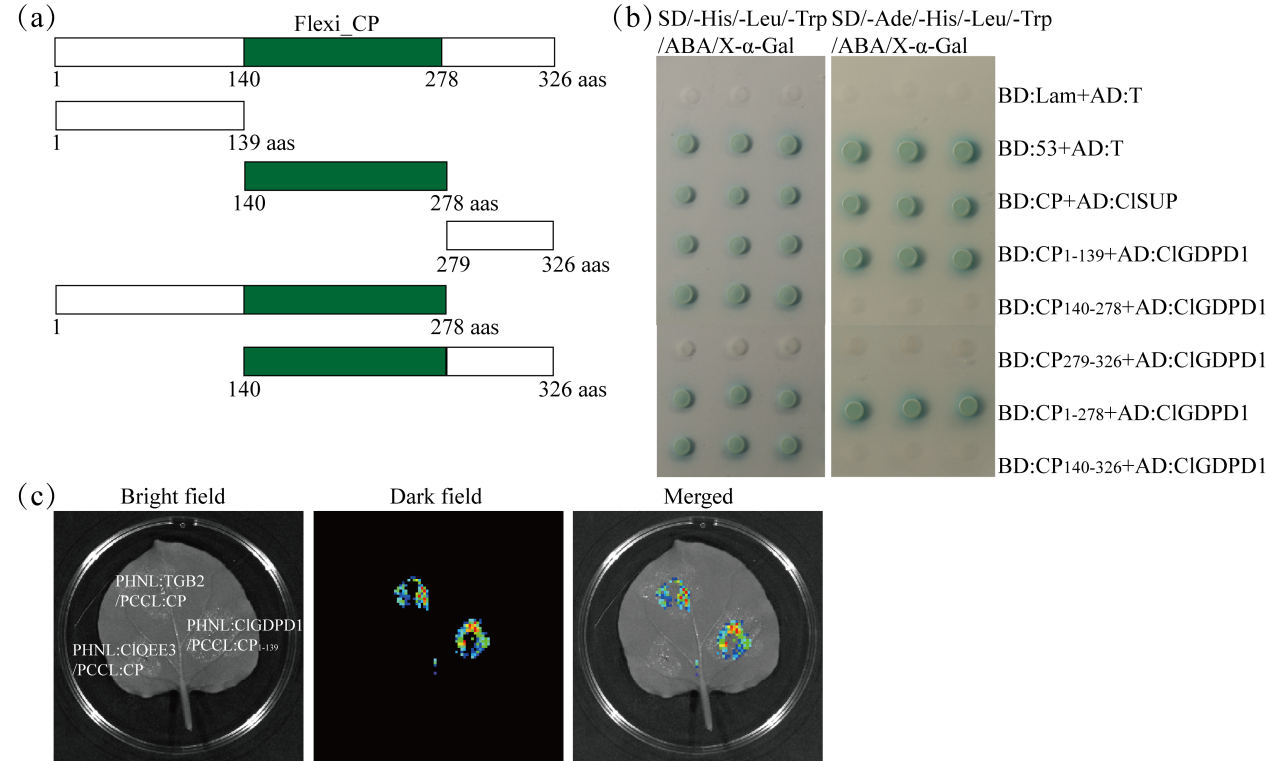


**Fig. S3 CP_1-139_ interacted with ClGDPD1.** (a) Schematic representation of CP predicted domains and five CP truncated mutants. (b) The yeast two-hybrid assay confirmed the interaction of truncated CP mutants with ClGDPD1. (C) A firefly luciferase complementation imaging assay confirms the interaction between CP_1-139_ and ClGDPD1. PHNL:TGB2/PCCL:CP, positive control; PHNL:ClOEE3/PCCL:CP, negative control.


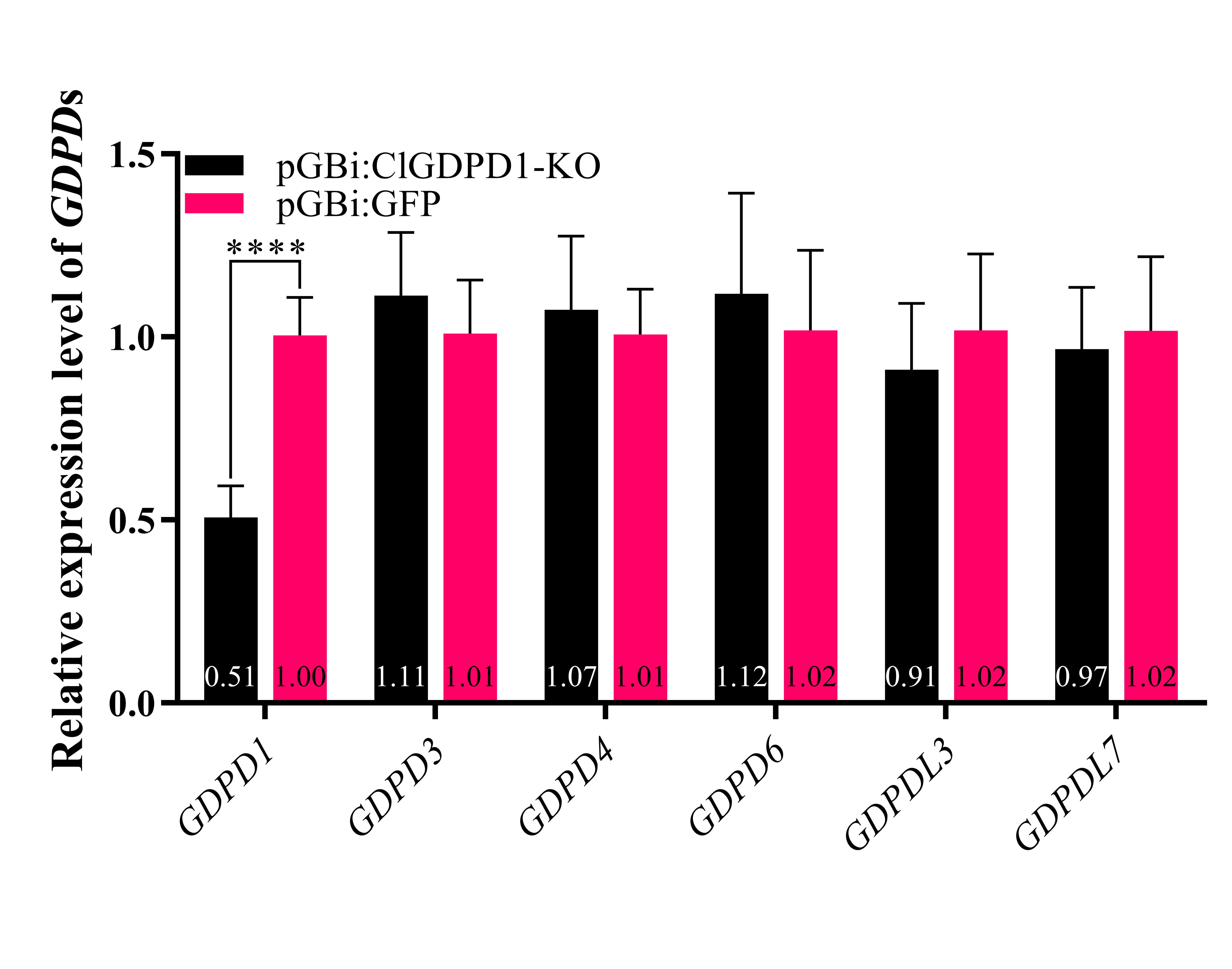


**Fig. S4 Silencing *ClGDPD1* in Eureka lemon leaves had no significant effect on the expression of other *GDPD*s.** *Actin* was used as an internal reference gene, t-test, n = 9, *****p*<0.0001.


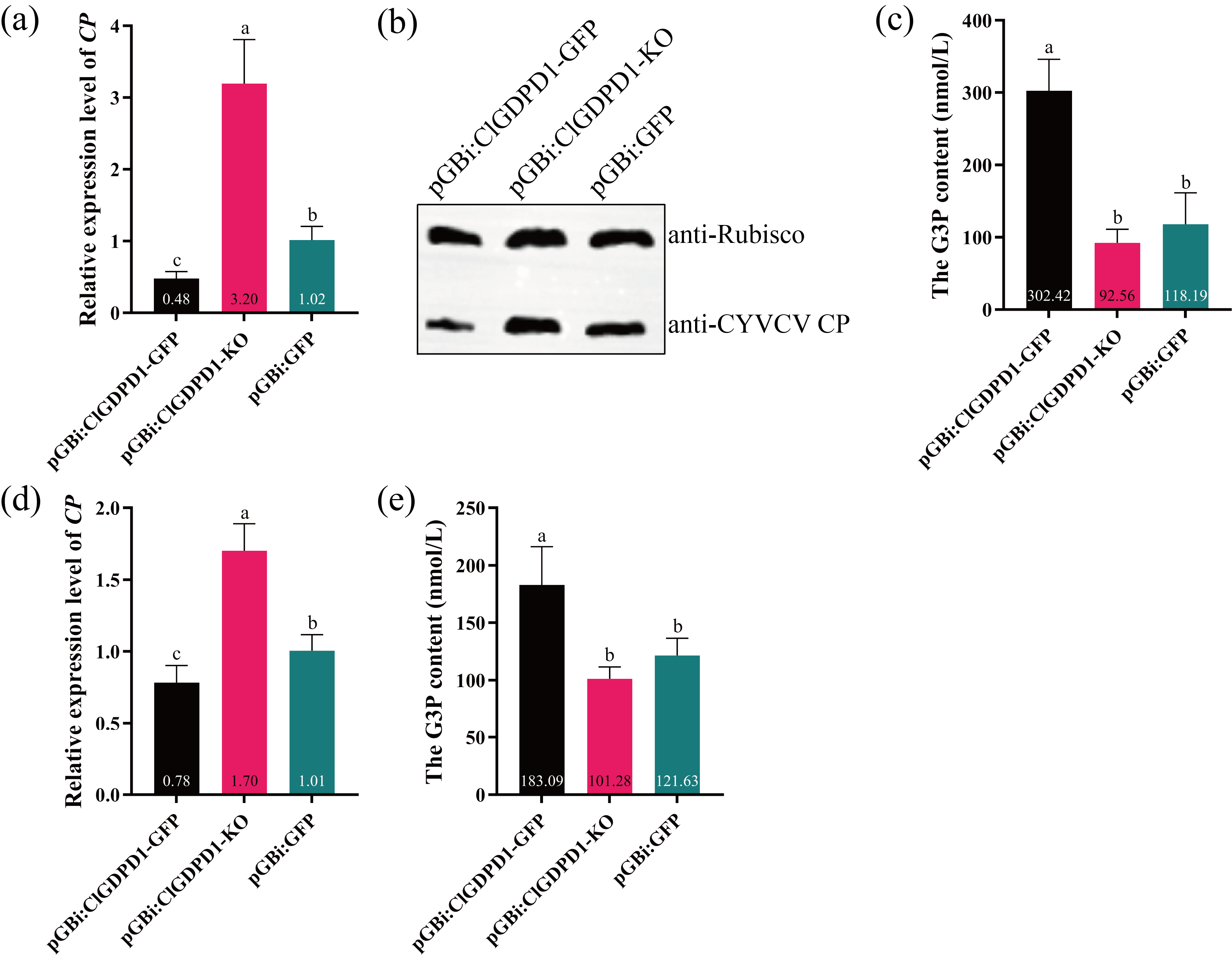


**Fig. S5 ClGDPD1 reduces CYVCV titer in CYVCV-infected Eureka lemon leaves.** (a) The relative expression of *CP* in CYVCV-infected Eureka lemon leaves which transient expression and silence of *ClGDPD1* at 8 days past-infiltration (dpi). *Actin* was used as an internal reference gene, one-way ANOVA test, *p*<0.05 indicates significance, n = 9. (b) Western blotting was used to assay the CYVCV CP content with an anti-CYVCV CP antibody. (c) The content of glycerol-3-phosphate (G3P) at 8 dpi. One-way ANOVA test, *p*<0.05 indicates significance, n = 8. (d) The relative expression of *CP* at 30 dpi. *Actin* was used as an internal reference gene, one-way ANOVA test, *p*<0.05 indicates significance, n = 9. (e) The content of G3P at 30 dpi. One-way ANOVA test, *p*<0.05 indicates significance, n = 8.


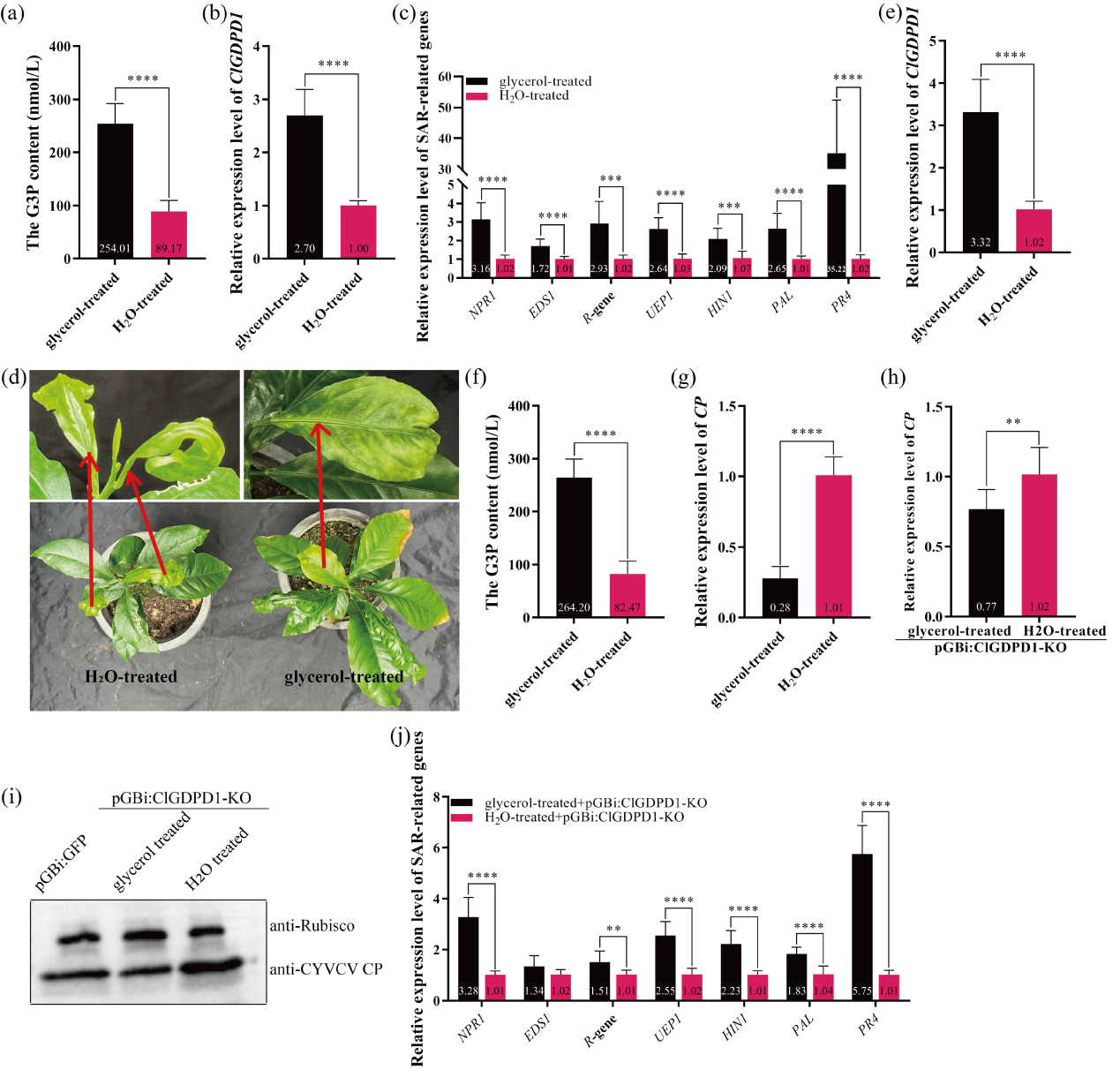


**Fig. S6 Glycerol treatment increased the glycerol-3-phosphate (G3P) content and positively regulated the plant resistance to CYVCV.** (a) The G3P content in glycerol treated virus-free Eureka lemon leaves. T-test, n = 8, *****p*<0.0001. (b-c) The expression of *ClGDPD1* and systemic acquired resistance (SAR)-related genes in glycerol treated virus-free Eureka lemon leaves. *Actin* was used as an internal reference gene, t-test, n = 9, ****p*<0.001, *****p*<0.0001. (d) The CYVCV-infected Eureka lemon symptoms at two months after glycerol treatment. (e) The expression of *ClGDPD1* in glycerol treated CYVCV-infected Eureka lemon leaves. *Actin* was used as an internal reference gene, t-test, n = 9, *****p*<0.0001. (f) The G3P content in glycerol treated CYVCV-infected Eureka lemon leaves. T-test, n = 8, *****p*<0.0001. (g) The expression of *CP* in glycerol treatment Eureka lemon leaves. *Actin* was used as an internal reference gene, t-test, n = 9, *****p*<0.0001. (h, j) The expression of *CP* and SAR-related genes in *ClGDPD1*-silenced young leaves which treatment with glycerol. *Actin* was used as an internal reference gene, t-test, n = 9, ***p*<0.01, *****p*<0.0001. (i) WB assay the CYVCV CP content using an anti-CYVCV CP antibody.

**
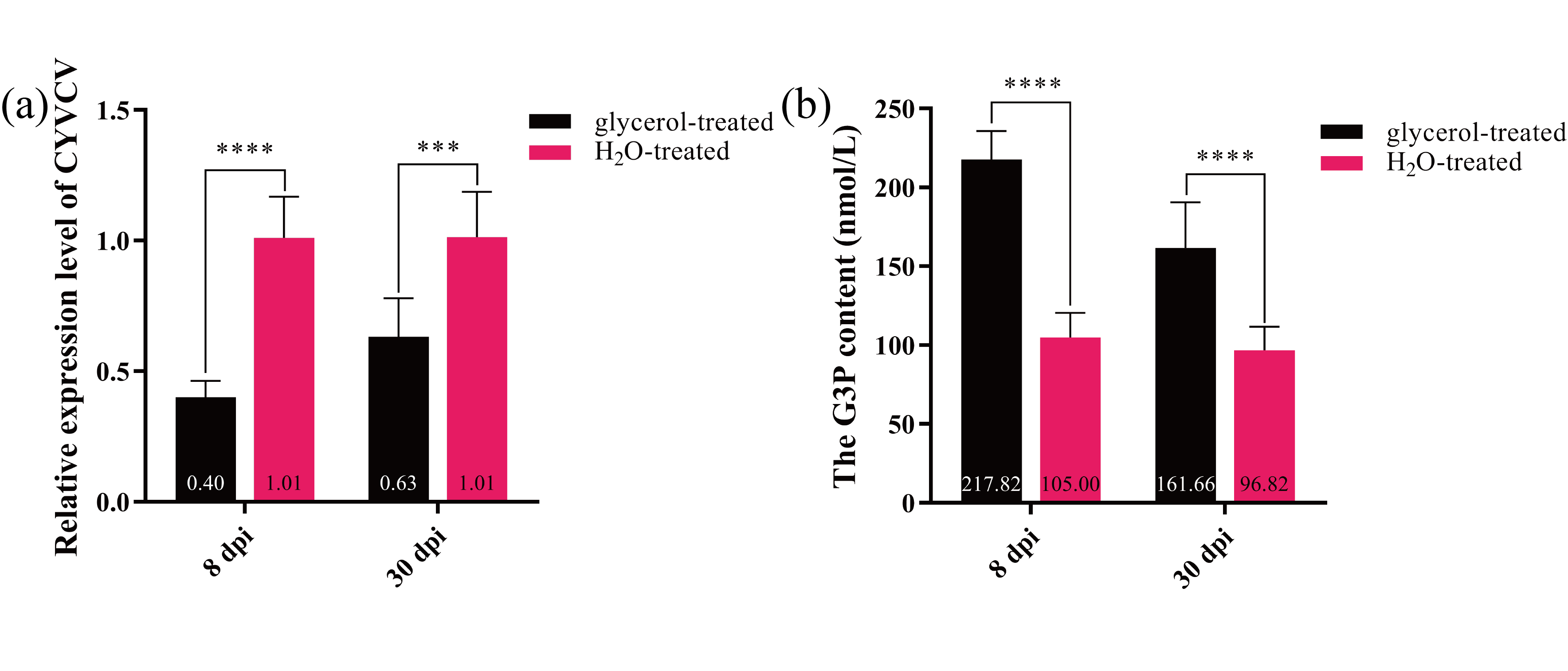
Fig. S7 Glycerol treatment reduced the CYVCV titer and increased the glycerol-3-phosphate (G3P) content.** (a) The CYVCV titer in glycerol-treated *ClGDPD1*-silenced CYVCV-infected Eureka lemon leaves at 8 and 30 dpi. *Actin* was used as an internal reference gene, t-test, n = 9, ****p*<0.001, *****p*<0.0001. (b) The G3P content in glycerol-treated *ClGDPD1*-silenced CYVCV-infected Eureka lemon leaves at 8 and 30 dpi. T-test, n = 8, *****p*<0.0001.


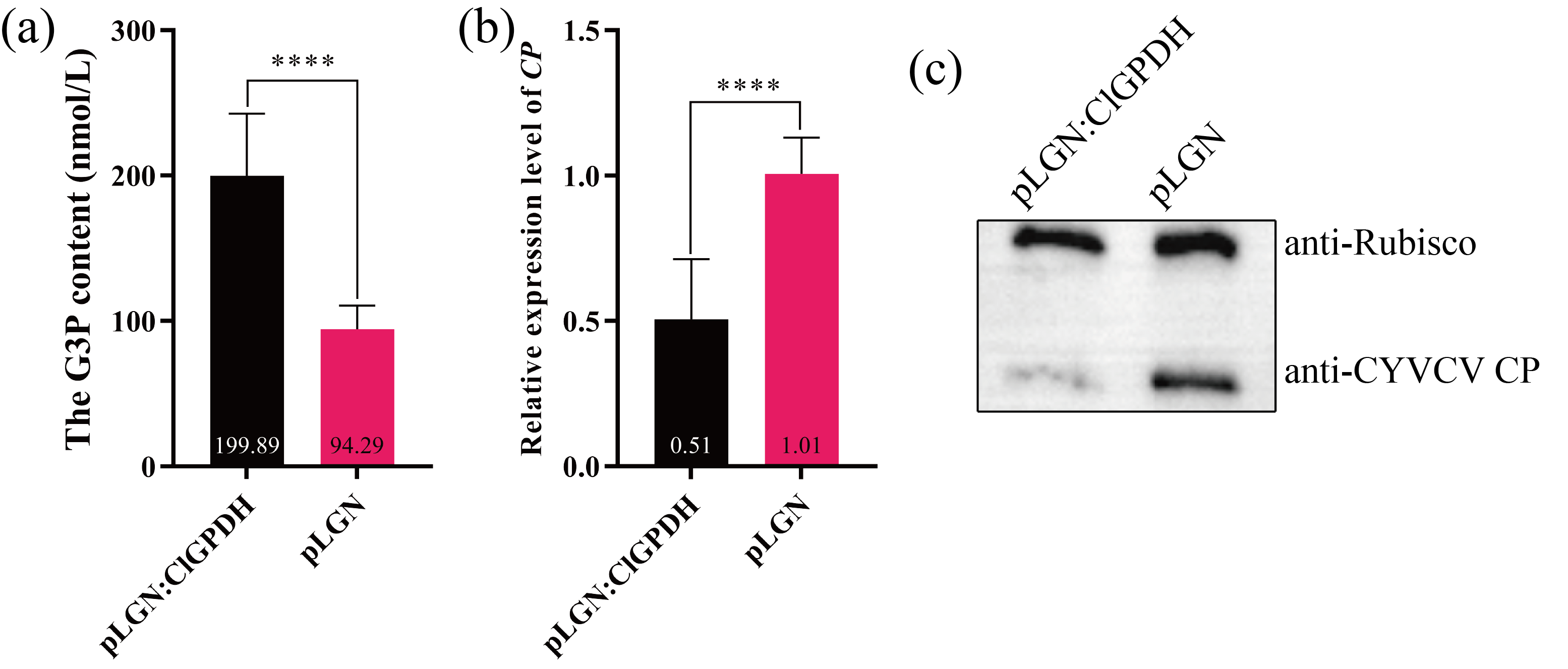


**Fig. S8 Over-expression of *ClGPDH* increased the glycerol-3-phosphate (G3P) content and positively regulated the plant resistance to CYVCV.** (a) The G3P content in *ClGPDH* transgenic hairy root. T-test, n = 8, *****p*<0.0001. (b-c) The CYVCV titer in the *ClGPDH* transgenic hairy root was determined using real-time quantitative PCR (RT-qPCR) and western blotting (WB) analysis. *Actin* was used as an internal reference gene, t-test, n = 9, *****p*<0.0001 for the RT-qPCR assay. An anti-CYVCV CP antibody was used for the WB assay.


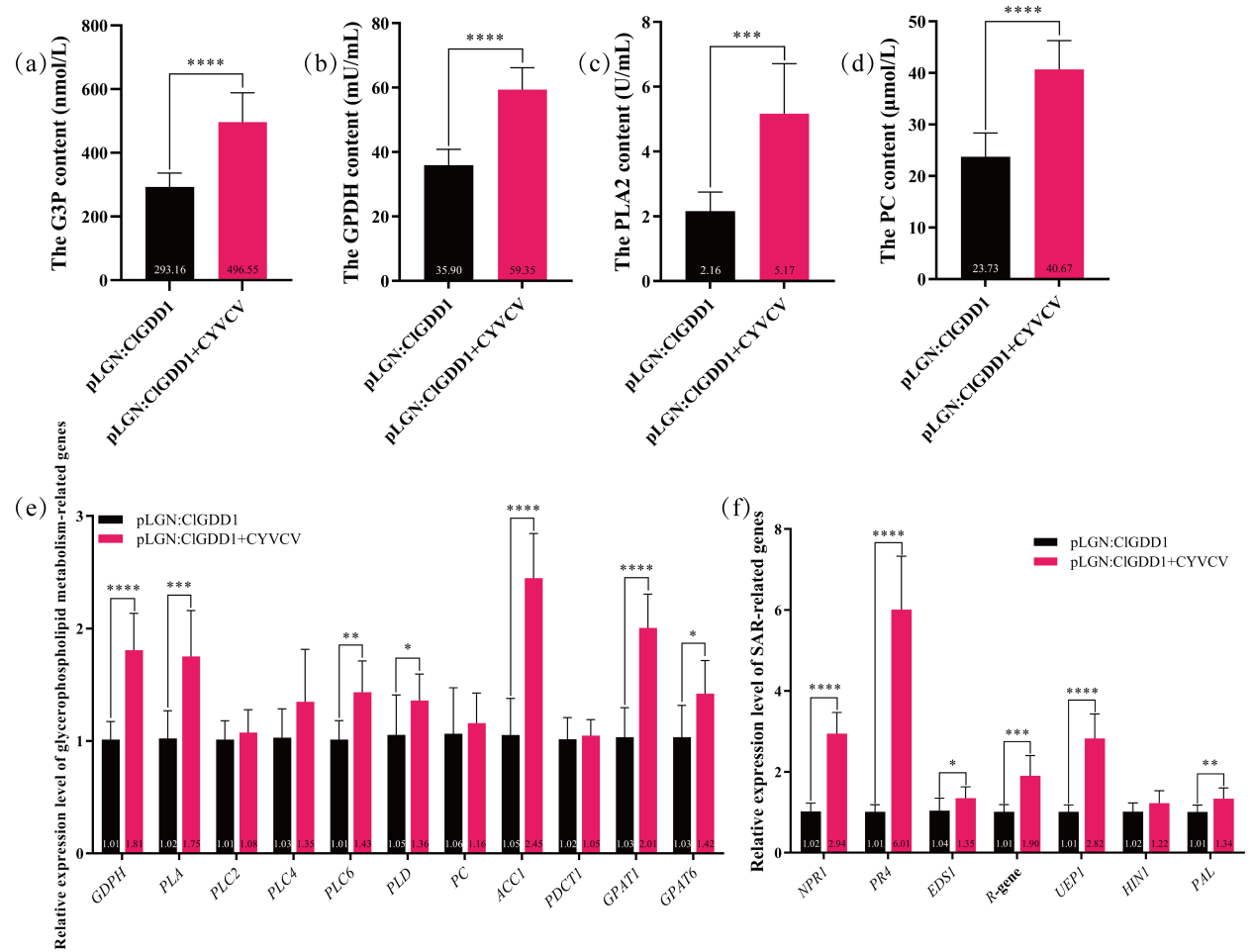


**Fig. S8 The glycerophosphate metabolism related substances and genes expression in virus-free and CYVCV-infected *ClGDPD1* transgenic Eureka lemon.** (a-d) The content of G3P, GPDH, PLA2, and PC in virus-free and CYVCV-infected *ClGDPD1* transgenic Eureka lemon. T-test, ****p*<0.001, *****p*<0.0001, n = 8. (e, f) The relative expression levels of genes related to glycerophosphate metabolism and systemic acquired resistance (SAR). *Actin* was used as an internal reference gene, t-test, n = 9, **p*<0.05, ***p*<0.01, ****p*<0.001, *****p*<0.0001.


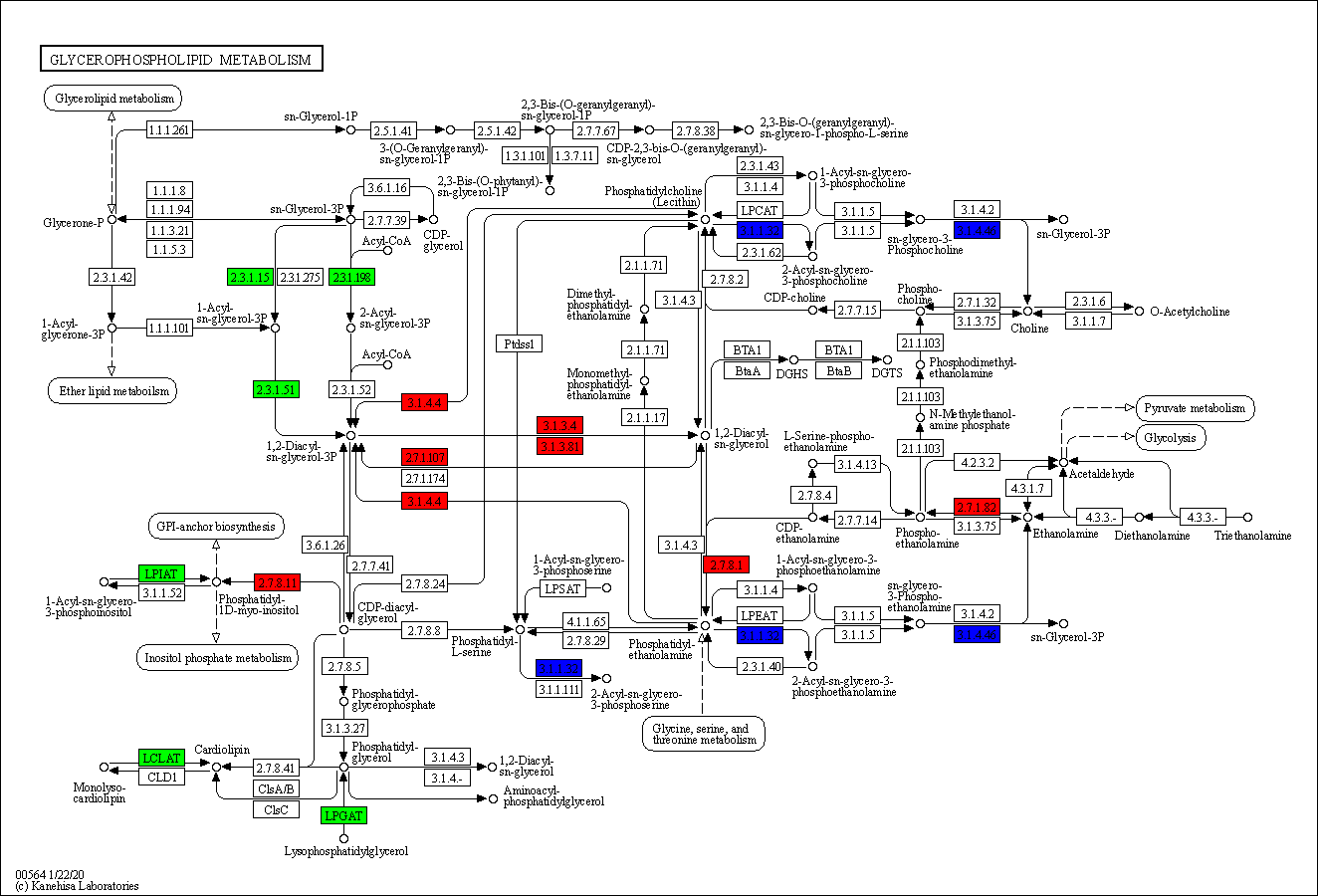


**Fig. S10 Differentially expressed genes enriched in glycerophospholipid metabolism.**

**Table S1** Pairwise alignment of GDPD1 protein sequences.

|  |  | 1 | 2 | 3 | 4 | 5 | 6 | 7 | 8 | 9 | 10 | 11 | 12 | 13 | 13 | 14 | 15 | 16 | 17 | 18 |
| --- | --- | --- | --- | --- | --- | --- | --- | --- | --- | --- | --- | --- | --- | --- | --- | --- | --- | --- | --- | --- |
| *Citrus australasica* Egl276330.1 | 1 | … | 98.97 | 99.48 | 99.74 | 99.48 | 98.97 | 99.74 | 99.23 | 99.74 | 98.45 | 99.23 | 98.48 | 98.97 | 76.55 | 73.97 | 79.95 | 80.93 | 81.12 | 76.29 |
| *Citrus clementina* XP_006447005 | 2 | 98.97 | … | 98.97 | 99.22 | 98.97 | 98.97 | 99.22 | 98.71 | 99.22 | 99.48 | 98.71 | 98.97 | 100.00 | 76.23 | 73.39 | 79.38 | 80.36 | 80.56 | 75.97 |
| *Citrus grandis* Cg2g010320.1 | 3 | 99.48 | 98.97 | … | 99.74 | 99.48 | 98.97 | 99.74 | 99.22 | 99.74 | 99.45 | 99.22 | 100.00 | 98.97 | 76.49 | 74.16 | 79.90 | 80.88 | 81.33 | 76.23 |
| *Citrus hongheensis* Chh232450.1 | 4 | 99.74 | 99.22 | 99.74 | … | 99.74 | 99.22 | 100.00 | 99.48 | 100.00 | 98.71 | 99.48 | 99.74 | 99.22 | 76.74 | 74.16 | 80.15 | 81.14 | 81.33 | 76.49 |
| *Citrus ichangensis* Cic186990.1 | 5 | 99.48 | 98.97 | 99.48 | 99.74 | … | 98.97 | 99.74 | 99.22 | 99.74 | 98.45 | 99.22 | 99.48 | 98.97 | 76.74 | 74.16 | 80.41 | 81.14 | 81.33 | 76.49 |
| *Citrus limon* WKB17708.1 | 6 | 98.97 | 98.97 | 98.97 | 99.22 | 98.97 | … | 99.22 | 98.71 | 99.22 | 98.45 | 98.71 | 98.97 | 98.97 | 75.97 | 73.39 | 79.38 | 80.62 | 80.56 | 75.71 |
| *Citrus linwuensis* LW231400.1 | 7 | 99.74 | 99.22 | 99.74 | 100.00 | 99.74 | 99.22 | … | 99.48 | 100.00 | 98.71 | 99.48 | 99.74 | 99.22 | 76.71 | 74.16 | 80.15 | 81.14 | 81.33 | 76.49 |
| *Citrus mangshanensis* Cms123180.1 | 8 | 99.23 | 98.71 | 99.22 | 99.48 | 99.22 | 98.71 | 99.48 | … | 99.48 | 98.19 | 98.97 | 99.22 | 98.71 | 76.49 | 73.90 | 79.64 | 81.14 | 80.82 | 76.23 |
| *Citrus medica* Cme125720.2 | 9 | 99.74 | 99.22 | 99.74 | 100.00 | 99.74 | 99.22 | 100.00 | 99.48 | … | 98.71 | 99.48 | 99.74 | 99.22 | 76.74 | 74.16 | 80.15 | 81.14 | 81.33 | 76.49 |
| *Citrus reticulata* Cre2g_022810.1 | 10 | 98.45 | 99.48 | 99.45 | 98.71 | 98.45 | 98.45 | 98.71 | 98.19 | 98.71 | … | 98.19 | 98.45 | 99.48 | 75.71 | 73.39 | 79.12 | 80.36 | 80.56 | 75.45 |
| *Poncirus trifoliata* Pt2g009590.1 | 11 | 99.23 | 98.71 | 99.22 | 99.48 | 99.22 | 98.71 | 99.48 | 98.97 | 99.48 | 98.19 | … | 99.22 | 98.71 | 76.74 | 74.42 | 80.15 | 81.14 | 81.33 | 76.49 |
| *Citrus sinensis* KAH9791820.1 | 12 | 98.48 | 98.97 | 100.00 | 99.74 | 99.48 | 98.97 | 99.74 | 99.22 | 99.74 | 98.45 | 99.22 | … | 98.97 | 76.49 | 74.16 | 79.90 | 80.88 | 81.33 | 76.23 |
| *Citrus meyerii* PP895201 | 13 | 98.97 | 100.00 | 98.97 | 99.22 | 98.97 | 98.97 | 99.22 | 98.71 | 99.22 | 99.48 | 98.71 | 98.97 | … | 76.22 | 73.39 | 79.38 | 80.36 | 80.56 | 75.97 |
| *Herrania umbratica* XP_021297951.1 | 14 | 76.55 | 76.23 | 76.49 | 76.74 | 76.74 | 75.97 | 76.74 | 76.49 | 76.74 | 75.71 | 76.74 | 76.49 | 76.22 | … | 78.74 | 77.46 | 77.49 | 77.63 | 96.59 |
| *Malus domestica* XP_008341262.2 | 15 | 73.97 | 73.39 | 74.16 | 74.16 | 74.16 | 73.39 | 74.16 | 73.90 | 74.16 | 73.39 | 74.42 | 74.16 | 73.39 | 78.74 | … | 77.72 | 76.70 | 77.89 | 79.79 |
| *Mangifera indica* XP_044502999.1 | 16 | 79.95 | 79.38 | 79.90 | 80.15 | 80.41 | 78.38 | 80.15 | 79.64 | 80.15 | 79.12 | 80.15 | 79.90 | 79.38 | 77.46 | 77.72 | … | 83.68 | 90.75 | 78.76 |
| *Melia azedarach* KAJ4729513.1 | 17 | 80.93 | 80.36 | 80.88 | 81.14 | 81.14 | 80.62 | 81.14 | 81.14 | 81.14 | 80.36 | 81.14 | 80.88 | 80.36 | 77.49 | 76.70 | 83.68 | … | 85.35 | 78.01 |
| *Pistacia vera* XP_031271470.1 | 18 | 81.12 | 80.56 | 81.33 | 81.33 | 81.33 | 80.56 | 81.33 | 80.82 | 81.33 | 80.56 | 81.33 | 81.33 | 80.56 | 77.63 | 77.89 | 90.75 | 85.35 | … | 77.89 |
| *Theobroma cacao* XP_007031952.2 | 19 | 76.29 | 75.97 | 76.23 | 76.49 | 76.49 | 75.71 | 76.49 | 76.23 | 76.49 | 75.45 | 76.49 | 76.23 | 75.97 | 96.59 | 79.79 | 78.76 | 78.01 | 77.89 | … |

**Table S2** The Eureka lemon *ClGPDH* hairy root transformation system.

|  | Sample number | Rooting number | Rooting rate % | Positive number | Detection rate % | Root number | Root length |
| --- | --- | --- | --- | --- | --- | --- | --- |
| pLGN:ClGPDH  pLGN  P value | 60  60 | 46  33 | 76.67%  55.00% | 36  17 | 78.26%  51.51% | 5.39±0.40  3.82±0.44  0.0207 | 6.38±0.29  5.52±0.50  0.1422 |

**Table S3** Sequencing data and sequence alignment with the *Citrus sinensis* reference genome.

| Samples | Clean Reads | Mapped Reads | Uniq Mapped Reads |
| --- | --- | --- | --- |
| CC1 | 43,829,540 | 38,170,130 (87.09%) | 36,670,019 (83.67%) |
| CC2 | 45,604,434 | 39,893,282 (87.48%) | 37,641,030 (82.54%) |
| CC3 | 47,360,184 | 42,223,338 (89.15%) | 40,623,885 (85.78%) |
| CC4 | 40,967,224 | 35,424,330 (86.47%) | 33,909,388 (82.77%) |
| CK1 | 40,971,406 | 36,561,046 (89.24%) | 35,077,493 (85.61%) |
| CK2 | 38,859,534 | 34,506,140 (88.80%) | 32,855,649 (84.55%) |
| CK3 | 40,559,684 | 36,095,770 (88.99%) | 34,788,630 (85.77%) |
| CK4 | 41,741,768 | 36,711,085 (87.95%) | 35,357,027 (84.70%) |

**Table S4** The relevant differentially expressed genes annotated in “plant-pathogen interaction” (ko04626), “phenylpropanoid biosynthesis” (ko00940), “mitogen-activated protein kinases (MAPK) signaling pathway-plant” (ko04016), and “plant hormone signal transduction” (ko04075).

Showed in XLS. worksheet Table S4

**Table S5** Primers used in this study.

| Usage | Primer Name | Sequence of prime 5ˊ-3ˊ |
| --- | --- | --- |
| Y2H | Y2H-CP-F | atggccatggaggccgaattcATGAGCTTCGACTACACTCACCC |
|  | Y2H-CP-R | ccgctgcaggtcgacggatccGATGTTGAAAGGGGTCGGGC |
|  | Y2H-ClGDPD1-F | gccatggaggccagtgaattcATGGCTCTTAAAGCTGTTCATGTCT |
|  | Y2H-ClGDPD1-R | cagctcgagctcgatggatccATGGTGTATTAATTCGGGGATTAGC |
| BiFC | BiFC-CP-F | tggcgcgccactagtggatccATGAGCTTCGACTACACTCACCC |
|  | BiFC-CP-R | agcggtaccctcgaggtcgacGATGTTGAAAGGGGTCGGGC |
|  | BiFC-ClGDPD1-F | tggcgcgccactagtggatccATGGCTCTTAAAGCTGTTCATGTCT |
|  | BiFC-ClGDPD1-R | agcggtaccctcgaggtcgacATGGTGTATTAATTCGGGGATTAGC |
| LCI | LCI-CP-F | acgggggacgagctcggtaccATGAGCTTCGACTACACTCACCC |
|  | LCI-CP-R | cgcgtacgagatctggtcgacGATGTTGAAAGGGGTCGGGC |
|  | LCI-ClGDPD1-F | acgggggacgagctcggtaccATGGCTCTTAAAGCTGTTCATGTCT |
|  | LCI-ClGDPD1-R | aacatcgtatgggtagtcgacATGGTGTATTAATTCGGGGATTAGC |
| Pull down | polldown-CP-1F | atggccatggaggccgaattcATGAGCTTCGACTACACTCACCC |
|  | polldown-CP-1R | acctagtataggggacatCGATCCTCCTCCGATGTTGAAAGGGGTCGGGC |
|  | polldown-CP-2F | gcccgacccctttcaacatcGGAGGAGGATCGATGTCCCCTATACTAGGTTATTGGAAA |
|  | polldown-CP-2R | ccgctgcaggtcgacggatccTTATTTTGGAGGATGGTCGCC |
|  | polldown-ClGDPD1-F | atggccatggaggccgaattcATGGCTCTTAAAGCTGTTCATGTCT |
|  | polldown-ClGDPD1-R | ccgctgcaggtcgacggatccATGATGATGATGATGATGCGATCCTCCTCCATGGTGTATTAATTCGGGGATTAGC |
| yeast two-hybrid (Y2H) assays of interacting domain(s) of ClGDPD1 | Y2H-ClGDPD1-1F | gccatggaggccagtgaattcATGGCTCTTAAAGCTGTTCATGTCT |
|  | Y2H-ClGDPD1-2F | gccatggaggccagtgaattcATGCTGGTGGTGGGACATAGAG |
|  | Y2H-ClGDPD1-3F | gccatggaggccagtgaattcATGCAAGAGATTACTGAGGCCG |
|  | Y2H-ClGDPD1-1R | cagctcgagctcgatggatccAAACTTCGGTATCCTAAACGACGC |
|  | Y2H-ClGDPD1-2R | cagctcgagctcgatggatccAACCAAATCGACAATCACCCC |
|  | Y2H-ClGDPD1-3R | cagctcgagctcgatggatccATGGTGTATTAATTCGGGGATTAGC |
| yeast two-hybrid (Y2H) assays of interacting domain(s) of CP | Y2H-CP_1_-F | atggccatggaggccgaattcATGAGCTTCGACTACACTCACCC |
|  | Y2H-CP_140_-F | atggccatggaggccgaattcATGACTAACTCCATAGCTAACCCTAAAC |
|  | Y2H-CP_279_-F | atggccatggaggccgaattcATGAGGATCGCAAATCAG |
|  | Y2H-CP_139_-R | ccgctgcaggtcgacggatccGACCGGTGAGTATCTTAGGTT |
|  | Y2H-CP_278_-R | ccgctgcaggtcgacggatccCTCCGCGGAAGTAGGGTGTC |
|  | Y2H-CP_326_-R | ccgctgcaggtcgacggatccGATGTTGAAAGGGGTCGGGC |
| BiFC of domain ClGDPD1 | BiFC-ClGDPD1-F | tggcgcgccactagtggatccATGCTGGTGGTGGGACATAGA |
|  | BiFC-ClGDPD1-R | agcggtaccctcgaggtcgacATGGTGTATTAATTCGGGGATTAGC |
| BiFC of domain CP | LCI-CP_1_-F | acgggggacgagctcggtaccATGAGCTTCGACTACACTCACCC |
|  | LCI-CP_139_-R | cgcgtacgagatctggtcgacGACCGGTGAGTATCTTAGGTT |
| Subcellular localization | CP-mCherry-1F | gcttcgaattctgcagtcgacATGAGCTTCGACTACACTCACCC |
|  | CP-mCherry-1R | cctcctcgcccttgctcaccatCGATCCTCCTCCGATGTTGAAAGGGGTCGGGC |
|  | CP-mCherry-2F | gcccgaggggtttcaacatcGGAGGAGGATCGATGGTGAGCAAGGGCGAGGAGG |
|  | CP-mCherry-2R | gcccttgctcaccatggatccTTACTTGTACAGCTCGTCCATGCCGCC |
|  | ClGDPD1-GFP-1F | gcttcgaattctgcagtcgacATGGCTCTTAAAGCTGTTCATGTCT |
|  | ClGDPD1-GFP-1R | gagaaaaagattagtcttcatCGATCCTCCTCCATGGTGTATTAATTCGGGGATTAGC |
|  | ClGDPD1-GFP-2F | GCTAATCCCCGAATTAATACACCATGGAGGAGGATCGATGAAGACTAATCTTTTTCTC |
|  | ClGDPD1-GFP-2R | gcccttgctcaccatggatccTTAGAGTTCGTCGTGTTTGTATAGTTCA |
| expression CP | pGBi-CP-F | actctagggactagtcccgggATGAGCTTCGACTACACTCACCC |
|  | pGBi-CP-R | tggatcctaggtgagtctagaTTAGATGTTGAAAGGGGTCGGG |
|  | pGBi-CP-GST-1F | actctagggactagtcccgggATGAGCTTCGACTACACTCACCC |
|  | pGBi-CP-GST-1R | acctagtataggggacatCGATCCTCCTCCGATGTTGAAAGGGGTCGGGC |
|  | pGBi-CP-GST-2F | gcccgacccctttcaacatcGGAGGAGGATCGATGTCCCCTATACTAGGTTATTGGAAA |
|  | pGBi-CP-GST-2R | tggatcctaggtgagtctagaTTATTTTGGAGGATGGTCGCC |
|  | pGBi-CP_1-139_-GST-1F | actctagggactagtcccgggATGAGCTTCGACTACACTCACCC |
|  | pGBi-CP_1-139_-GST-1R | acctagtataggggacatCGATCCTCCTCCGACCGGTGAGTATCTTAGGTT |
|  | pGBi-CP_1-139_-GST-2F | aacctaagatactcaccggtcGGAGGAGGATCGATGTCCCCTATACTAGGTTATTGGAAA |
|  | pGBi-CP_1-139_-GST-2R | tggatcctaggtgagtctagaTTATTTTGGAGGATGGTCGCC |
|  | pGBi-CP_140-326_-GST-1F | actctagggactagtcccgggATGACTAACTCCATAGCTAACCCTAAAC |
|  | pGBi-CP_140-326_-GST-1R | acctagtataggggacatCGATCCTCCTCCGATGTTGAAAGGGGTCGGGC |
|  | pGBi-CP_140-326_-GST-2F | gcccgacccctttcaacatcGGAGGAGGATCGATGTCCCCTATACTAGGTTATTGGAAA |
|  | pGBi-CP-GST-2R | tggatcctaggtgagtctagaTTATTTTGGAGGATGGTCGCC |
| expression ClGDPD1 | pGBi-ClGDPD1-1F | actctagggactagtcccgggATGGCTCTTAAAGCTGTTCATGTCT |
|  | pGBi-ClGDPD1-1R | gagaaaaagattagtcttcatCGATCCTCCTCCATGGTGTATTAATTCGGGGATTAGC |
|  | pGBi-ClGDPD1-2F | GCTAATCCCCGAATTAATACACCATGGAGGAGGATCGATGAAGACTAATCTTTTTCTC |
|  | pGBi-ClGDPD1-2R | tggatcctaggtgagtctagaTTAGAGTTCGTCGTGTTTGTATAGTTCA |
| expression domain ClGDPD1 | pGBi-ClGDPD1-1-47-1F | actctagggactagtcccgggATGGCTCTTAAAGCTGTTCATGTCT |
|  | pGBi-ClGDPD1-1-47-1R | gagaaaaagattagtcttcatCGATCCTCCTCCAAACTTCGGTATCCTAAACGACGC |
|  | pGBi-ClGDPD1-1-47-2F | GCGTCGTTTAGGATACCGAAGTTTGGAGGAGGATCGATGAAGACTAATCTTTTTCTC |
|  | pGBi-ClGDPD1-1-47-2R | tggatcctaggtgagtctagaTTAGAGTTCGTCGTGTTTGTATAGTTCA |
|  | pGBi-ClGDPD1-48-328-1F | actctagggactagtcccgggATGCTGGTGGTGGGACATAGAG |
|  | pGBi-ClGDPD1-48-328-1R | gagaaaaagattagtcttcatCGATCCTCCTCCAACCAAATCGACAATCACCCC |
|  | pGBi-ClGDPD1-48-328-2F | GGGGTGATTGTCGATTTGGTTGGAGGAGGATCGATGAAGACTAATCTTTTTCTC |
|  | pGBi-ClGDPD1-48-328-2R | tggatcctaggtgagtctagaTTAGAGTTCGTCGTGTTTGTATAGTTCA |
|  | pGBi-ClGDPD1-329-386-1F | actctagggactagtcccgggATGCAAGAGATTACTGAGGCCG |
|  | pGBi-ClGDPD1-329-386-1R | gagaaaaagattagtcttcatCGATCCTCCTCCATGGTGTATTAATTCGGGGATTAGC |
|  | pGBi-ClGDPD1-329-386-2F | GCTAATCCCCGAATTAATACACCATGGAGGAGGATCGATGAAGACTAATCTTTTTCTC |
|  | pGBi-ClGDPD1-329-386-2R | tggatcctaggtgagtctagaTTAGAGTTCGTCGTGTTTGTATAGTTCA |
| silence ClGDPD1 | pGBi-ClGDPD1-F | actctagggactagtcccgggATGTAATGGGTCTGTTCTCAGCAA |
|  | pGBi-ClGDPD1-R | tggatcctaggtgagtctagaAGAGGATTACAGAGCTCTCTTTATCAGA |
| ClGDPD1 transgenic Eureka lemon | pLGN-ClGDPD1-F | ggacagggtacccggggatccATGGTGTATTAATTCGGGGATTAGC |
|  | pLGN-ClGDPD1-R | tctcattaaagcagggaattcATGGTGTATTAATTCGGGGATTAGC |
| ClGPDH root transgenic Eureka lemon | pLGN-ClGDPH-F | ggacagggtacccggggatccATGCGTCTTCTTTCTTCTTTATATTTG |
|  | pLGN-ClGDPH-R | tctcattaaagcagggaattcGTAGTACTGAGTAGAGCCTTCCAG |
| transgenic plant detection | GUS-F | CGCGGTTTTTCACCGAAGTT |
|  | GUS-R | GCACGGGAATATTTCGCCAC |
| All of the primers used for qPCR | Actin-F | CATCCCTCAGCACCTTCC |
|  | Actin-R | CCAACCTTAGCACTTCTCC |
|  | ClNPR1-qF | AGAGGACCCAAGTTTGAGCT |
|  | ClNPR1-qR | CAGGCATCATCATCCACAC |
|  | ClPR4-qF | CTACCACCCTGAGCAAAT |
|  | ClPR4-qR | ACACTCCTTCGTCCAAAT |
|  | ClEDS1-qF | GATCCAGCGGGGAATCATGT |
|  | ClEDS1-qR | TCCACCACTTGACGAAGGTC |
|  | ClR-qF | TTAAAGAAGATCCGCGGCGT |
|  | ClR-qR | TGAGACACAGACACCCAAGC |
|  | ClUEP1-qF | GGTCGAATCATCGGACACCA |
|  | ClUEP1-qR | GGACGAGGTGGAGTGTTGAC |
|  | ClHIN1-qF | CGCCAACAAAGCACTACCAC |
|  | ClHIN1-qR | GAGGCCAAGGATCAACACGA |
|  | PAL-qF | CAAGCGGGTTTCGGGTTC |
|  | PAL-qR | ACAGAGCAAGGTTGTTAGCG |
|  | GDPDL7-qF | CCTACGGTTCTATTCTCAA |
|  | GDPDL7-qR | CTTCGGGCTCAAAGTTAT |
|  | GDPDL3-qF | TGTCACAGAGCCACCTTT |
|  | GDPDL3-qR | GGAGCATAGCCATAGTCG |
|  | GDPD3-qF | AAAGGGTTACGGACATT |
|  | GDPD3-qR | ATGCGTGAGTTCTTCCT |
|  | GDPD6-qF | AGAAACTGCTGCTGCCTAC |
|  | GDPD6-qR | ACCTCTGCTTCGCCCTC |
|  | GDPD4-qF | CTTGTTGGCTCTTCACG |
|  | GDPD4-qR | AAGGCAGTTGTAGCATTTT |
|  | GDPD1-qF | TTATCTCATCAGGGTCATT |
|  | GDPD1-qR | CAGCCTCCTCCAACGA |
|  | GDPH-qF | TTGCTAAGAACGAAGGGAA |
|  | GDPH-qR | CTATGGCTGACGGTGGG |
|  | PLC4-qF | ATAAGTATGCGGAAGGTG |
|  | PLC4-qR | GTAATGAGACAACGGAGC |
|  | PLC2-qF | TTTTGATGGAGGAGTTGA |
|  | PLC2-qR | CCCGATTAGTGGGTTGTA |
|  | PLC6-qF | CTGCCGATAAAGTACGAC |
|  | PLC6-qR | TCCATTGGCTCTAAACAT |
|  | PLD-qF | TGGAGAAAGCAGGGTGGA |
|  | PLD-qR | CTCTGGGAAGCCAAATGC |
|  | PLA-qF | TCGGCAGAGCAATAGGA |
|  | PLA-qR | GGGAGGCAATGGTAATC |
|  | PC-qF | AGGTTGTCTTCGGTCTG |
|  | PC-qR | TTCCCATCGCTGTTTAG |
|  | ACC1-qF | GTCAGAAGCCCAAAGGAG |
|  | ACC1-qR | CCAAGGGAACGACAGAAT |
|  | NPK1-F | CAACGGGTCATCCAAGAGGT |
|  | NPK1-R | AGTCTTCTGCTTGGTAGCCG |
|  | CP-qF | TCCAACTCACAAACCCAGTC |
|  | CP-qR | ATGGGCTCTTGGTTTTCCTT |
|  | GPAT6-qF | CGGGAGTTCTTGTCGGAGAG |
|  | GPAT6-qR | ACGGTTGAAACTGAAAGCGG |
|  | FLS2-qF | TTGGCACCAGAGTTTGCGTA |
|  | FLS2-qR | TCTCCTCATCCAGTCCCGTT |
|  | RBOHD-qF | GAGCTTCCAAGAAAAGCGCC |
|  | RBOHD-qR | ACTGCGACAGAGTCATCACG |
|  | PDCT1-qF | GGTTCTGGGAATGGGAGAGC |
|  | PDCT1-qR | GTGTACTCGACGCCCATGAA |
|  | GPAT1-qF | GGACCCTGTTTTCCTCAGCA |
|  | GPAT1-qR | CTTCCGGGCAAACAACCAAG |
